# Odiparcil, a potential glycosaminoglycans clearance therapy in mucopolysaccharidosis VI—Evidence from *in vitro* and *in vivo* models

**DOI:** 10.1371/journal.pone.0233032

**Published:** 2020-05-15

**Authors:** Eugeni Entchev, Ingrid Jantzen, Philippe Masson, Stephanie Bocart, Bruno Bournique, Jean-Michel Luccarini, Andre Bouchot, Olivier Lacombe, Jean-Louis Junien, Pierre Broqua, Mireille Tallandier

**Affiliations:** 1 Inventiva Pharma, Bourgogne-Franche-Comté, Daix, France; 2 DImaCell Core Facility, Inserm UMR1231 CellImaP site, Université de Bourgogne, Bourgogne-Franche-Comté, Dijon, France; UMR 7365 CNRS UL, FRANCE

## Abstract

Mucopolysaccharidoses are a class of lysosomal storage diseases, characterized by enzymatic deficiency in the degradation of specific glycosaminoglycans (GAG). Pathological accumulation of excess GAG leads to multiple clinical symptoms with systemic character, most severely affecting bones, muscles and connective tissues. Current therapies include periodic intravenous infusion of supplementary recombinant enzyme (Enzyme Replacement Therapy–ERT) or bone marrow transplantation. However, ERT has limited efficacy due to poor penetration in some organs and tissues. Here, we investigated the potential of the β-D-xyloside derivative odiparcil as an oral GAG clearance therapy for Maroteaux–Lamy syndrome (Mucopolysaccharidosis type VI, MPS VI). *In vitro*, in bovine aortic endothelial cells, odiparcil stimulated the secretion of sulphated GAG into culture media, mainly of chondroitin sulphate (CS) /dermatan sulphate (DS) type. Efficacy of odiparcil in reducing intracellular GAG content was investigated in skin fibroblasts from MPS VI patients where odiparcil was shown to reduce efficiently the accumulation of intracellular CS with an EC_50_ in the range of 1 μM. *In vivo*, in wild type rats, after oral administrations, odiparcil was well distributed, achieving μM concentrations in MPS VI disease-relevant tissues and organs (bone, cartilage, heart and cornea). In MPS VI *Arylsulphatase B* deficient mice (*Arsb*^*-*^), after chronic oral administration, odiparcil consistently stimulated the urinary excretion of sulphated GAG throughout the treatment period and significantly reduced tissue GAG accumulation in liver and kidney. Furthermore, odiparcil diminished the pathological cartilage thickening observed in trachea and femoral growth plates of MPS VI mice. The therapeutic efficacy of odiparcil was similar in models of early (treatment starting in juvenile, 4 weeks old mice) or established disease (treatment starting in adult, 3 months old mice). Our data demonstrate that odiparcil effectively diverts the synthesis of cellular glycosaminoglycans into secreted soluble species and this effect can be used for reducing cellular and tissue GAG accumulation in MPS VI models. Therefore, our data reveal the potential of odiparcil as an oral GAG clearance therapy for MPS VI patients.

## Introduction

MPS VI (Maroteaux-Lamy syndrome) is caused by deficiency of the enzyme N-acetylgalactosamine-4-sulfatase (arylsulfatase B) [1–5]. This enzyme is required for lysosomal degradation of chondroitin sulphate type of GAG (CSGAG) consisting of CS and DS. Thus, the primary pathology in MPS VI disease is impaired degradation of cellular and tissue CS/ DS resulting in tissue GAG accumulation and leading to various clinical symptoms. The onset and severity of the disease might vary depending on the mutation and residual activity of the affected enzyme. MPS VI patients show the common MPS clinical disease manifestations including skeletal abnormalities (short stature, curved spine, restrictions of joints movement) [6,7], heart disease (valvular and myocardium thickening) [8–10], visual system failures (corneal clouding and glaucoma) [11,12] but lack neurological phenotypes [3–5].

In recent years tremendous advancements have been made in developing and applying therapies for MPS disease treatment and management [5,13,14]. These novel therapies aim to compensate for the lack of endogenous enzyme activity. They include enzyme replacement therapy, haematopoietic stem cells transplantation, gene therapies and stop codon read through. The most common clinical options for MPS VI treatment are bone marrow transplantation and enzyme replacement therapies (ERT). Due to the associated morbidity risks, bone marrow transplantation has been of limited use for MPS VI treatment [5]. ERT with galsulfase is more commonly used and consists of the intravenous infusion of exogenous recombinant enzymes to compensate for insufficient endogenous functions [5,15,16]. These therapies improve clinical manifestations of the disease (such as reduced mobility, endurance and growth) and are reported to slow down disease progression. However, ERT do not stop the progression of disease and patients might require additional, usually surgical, interventions for managing the most severe disease symptoms, such as cardiac valve replacement or corneal transplantation [9,17]. Gene therapies are providing promising new directions in investigating treatments for various MPS types, despite long-term safety concerns [14]. Because of low tissue distribution of exogenous recombinant enzymes (in bones, cartilage, eyes) and / or associated risks due to various immune responses or off-target gene effects, there remains a high unmet medical need, requiring further research and development of new therapies for MPS.

Here, we investigated the potential of odiparcil, a β-D-xyloside analog [18], as an oral GAG clearance therapy in MPS VI. β-D-xylosides are substrates of galactosyltransferase I (β4GalT7) [19–22], the enzyme responsible for the attachment of a galactosyl molecule to protein bound xylose that is required for the synthesis of O-glycosylated proteoglycans [23,24]. β-D-xyloside derivatives are found to be more efficient than D-xylose as substrates for β4GalT7, thus eliminating the need for core protein and xylosyltransferase but still engaging the downstream GAG synthesis machinery [19–22]. The newly synthesized β-D-xyloside-bound GAG (in case of odiparcil mainly CSGAG) are soluble and readily releasable into the circulatory system [25] and, therefore, might lead to a net reduction of cellular GAG [26–28]. Since MPS VI pathologies are caused by the cellular accumulation of CSGAG, it could be hypothesised that a reduction in CS/DS levels could provide relief from disease symptoms. Thus, odiparcil, might provide an oral GAG clearance therapeutic option for different MPS types. More specifically, by driving endogenous GAG synthesis towards circulating GAG, odiparcil would prevent or reduce the lysosomal and cellular accumulation of non-metabolized endogenous substrates observed in MPS. Our study validates this concept, *in vitro* in fibroblasts from MPS VI patients and *in vivo* in a murine model of MPS VI.

## Materials and methods

### Odiparcil and chemicals

Odiparcil (chemical name 4-methyl-7-(5-thio- β-D-xylopyranosyloxy)-2 H-chromen-2-one) was synthetized either at Inventiva (for *in vitro* and *in vivo* studies) or at Dr. Reddy’s Laboratories, India (for *in vivo* studies). All chemicals were purchased from Sigma Aldrich unless otherwise indicated.

### Analysis of secreted GAG from BAE cells after odiparcil treatment

GAG secreted into cell culture supernatant were analysed in Bovine aortic endothelial cells (ECACC 92010601), cultured in 6-well plates and incubated for 24 h in the presence of [^35^S] sodium sulphate (10 μCi/ml) and odiparcil solubilized in DMSO at various concentrations (1–10 μM; 0.1% final concentration of DMSO). The culture supernatants were recovered and the unincorporated [^35^S] was then removed by gel filtration on Sephadex G25 columns, the GAG being eluted in the column exclusion fraction (V0). A solution of cetylpyridinium chloride (0.1% final concentration) was added to the eluent in order to precipitate the GAG for 24 h at room temperature. The samples were then centrifuged and the supernatant was removed. The precipitate obtained was re-suspended in 2 M magnesium chloride and the GAG were precipitated with 5 volumes of 95% ethanol. After centrifugation, the alcoholic precipitates were re-suspended in 0.9% sodium chloride and then the radioactivity was measured.

In order to sort the GAG produced in the supernatants from cells in culture, the re-suspended alcoholic precipitates were treated with chondroitinase ABC (*Proteus vulgaris*) in a proportion of 0.5 mU/μL, for 3 h at 37°C. After inactivation of the enzyme for 3 min at 100°C, the undigested GAG were precipitated with 5 volumes of 95% ethanol, overnight at 4°C. After centrifugation, the alcoholic precipitates were re-suspended in 0.9% sodium chloride and then the radioactivity was measured on an aliquot fraction after addition of scintillation fluid in counting vials. GAG of heparan sulphate type were treated with heparinase II (*Flavobacterium heparinum*) in a proportion of 4 mU/μl, for 12 h at 30°C. After inactivation of the enzyme for 3 min at 100°C, the undigested GAG were precipitated with 5 volumes of 95% ethanol, overnight at 4°C. After centrifugation, the alcoholic precipitates were re-suspended in 0.9% sodium chloride and then the radioactivity was measured on an aliquot fraction after addition of scintillation fluid in counting vials.

### Cell culture and cells treatment

Primary skin fibroblasts from MPS VI patients GM00538 and GM02572 were obtained from the NIGMS Human Genetic Cell Repository at the Coriell Institute for Medical Research. Cell culturing was done according to the conditions provided by the Coriell Institute. For evaluation of the effect of odiparcil, cells were seeded in 96 well imaging plates (Falcon Cat N 353219) and two types of treatment were evaluated: i) prophylactic conditions—cells were treated with odiparcil before reaching confluence, 24 hours after plating; and ii) curative conditions—cells were treated with odiparcil after reaching confluence (10 days culturing). Cells were treated by odiparcil added to the culture media. This was done by replacing the initial culture media by media containing 0.1% DMSO with or without odiparcil (at 0.01, 0.03, 0.1, 0.3, 1, 3 and 10 μM). For treatment, the cells were incubated for 72 hours at 37°C, 5% CO_2_. All experimental conditions were performed in triplicate, i.e., 3 wells/test condition.

### Immunofluorescence and imaging

After odiparcil treatment as described above, culture media were removed to be used for dimethylmethylene blue (DMMB) assay and the cells were fixed in PFA 4%. Immunofluorescent staining was done following standard protocol comprising permeabilization with 0.1% Triton X 100, blocking with 2% NGS, staining with primary antibody and staining with secondary antibody and Hoechst (for nuclei visualization). For intracellular CS staining, before permeabilization the plates were treated with chondroitinase ABC (from *Proteus vulgaris*) at 0.1 U/ml in digestion buffer (Tris HCl 50 mM, Sodium acetate 60 mM, pH = 8) for 2 hours at 37°C. After digestion with chondroitinase ABC, cells were processed with the antibody staining protocol. The following antibodies were used: 1/200 anti-chondroitin sulphate [CS-56] (ab11570 Abcam), 1/200 anti-GLG1 (Golgi) antibody (ab103439 Abcam), 1/400 goat anti-mouse GAM-Alexa 488 (A11029 Invitrogen), 1/400 goat anti-mouse Alexa 546 (A11030 Invitrogen). Lysosomes were labelled with LAMP-1-GFP marker after transfection of primary skin fibroblast (GM00538) with the plasmid RC100016 (Origene). Transfection was performed with 2 ng DNA and using Amaxa Basic Nucleofector kit for primary fibroblasts (VPI-1002, Lonza). The cells were fixed 48 hours after transfection. Image acquisition was carried out using ImageXpress Micro (Molecular Devices).

### Animal studies

#### Test animals

The studies were conducted under EU animal welfare regulation for animal use in experimentation (European Directive 2010/63/EEC). The experimental protocols were submitted for approval by the Inventiva ethics committee “Comité de reflexion Ethique en Expérimentation Animale (CR2EA)” (registered by the “Ministère de l’Enseignement Supérieur et de la Recherche” under No. 104). All the procedures described below were reviewed and approved by the Inventiva ethics committee. Inventiva is fully accredited AAALAC company.

MPS VI model nonsense mutation *Arsb*^*m1J*^ homozygous mice [29,30] (referred to as *Arsb*^*-*^ throughout the manuscript) and WT littermates were obtained from internal breeding of stock mice derived from The Jackson Laboratory strain No: 005598 (C57BL/6J-Arsb^m1J^/GrsrJ). Genotyping and *Arsb*^*m1J*^ mutation identification were performed according to the protocol provided by The Jackson Laboratory. Each study group contained mix of male and female: 6 animals in disease progression studies and between 8 and 12 animals in odiparcil treatment studies.

Male Sprague Dawley rats (weight 250–300 g) for odiparcil tissue exposure study were purchased from Janvier Labs, France. The study was done with 12 animals.

Housing conditions. The animals were housed in groups of 3–10 (for mice) or 2 (for rats) in polypropylene cages (floor area = 1032 cm^2^) under standard conditions: room temperature (22 ± 2°C), hygrometry (55 ± 10%), light/dark cycle (12h/12h), air replacement (15–20 volumes/hour), water and food ad libitum. Mice were allowed to habituate for at least 5 days prior to experimentation. Mice were numbered by marking their tail using indelible markers. For the early disease model (treatment starting in juvenile, 4 weeks old mice), odiparcil administration in the weeks 1 to 6 was done per gavage with odiparcil in Methylcellulose 400 cps 1% / Poloxamer 188 0.1% / Water, freshly prepared every week. Later on, administration was per chow diet with odiparcil mixed before diet granulation. For advanced disease treatment (treatment starting in adult, 3 months old mice), administration was per diet from the beginning of the study. WT and *Arsb*^*-*^ animals were randomized into groups: control and two doses of odiparcil: 1.5 g odiparcil per kg of diet and 4.5 g odiparcil per kg of diet. For urine collection, animals were placed for 24 hours in standard metabolism cages (2 or 3 animals per cage). At the end of the studies, animals were anesthetized (O_2_ / isoflurane), blood for smears was collected from ocular sinus and after euthanasia samples from liver and kidney (snap frozen for total GAG analysis by Blyscan method or fixed for histology, see below) and from trachea and knee from the left hind leg were collected.

#### Odiparcil tissue exposure

Sprague Dawley rats were orally administered with odiparcil for 5-days, three times a day (t.i.d) with a 25 mg/kg/dose as a suspension in Methylcellulose 400 cps 1% / Poloxamer 188 0.1% / Water. On day 5, only one dose was administered and tissues–bone from head of femurs and cartilage also from head of femurs, cornea and heart–were collected at 30 min post-dosing, 3 animals per sample. Tissues were snap-frozen immediately after collection and stored at -20°C until odiparcil quantification by LC-MS/MS. Non compartmental method was applied with Phoenix WinNonLin^®^ to estimate the Cmax and AUC parameters. Tissue partition coefficient (Kp) was calculated on AUC values (AUC tissue / AUC plasma). Moreover, Kp for the heart was corrected for the significant blood content (26%) while it was not corrected for bone, cartilage and cornea.

#### GAG quantification

Total sulphated GAG in cell culture media were determined colorimetrically by DMMB method (described in [31] adapted for cell culture media). Total sulphated GAG in snap frozen liver and kidney samples and urine were measured using Blyscan assay from Biocolor Company (UK) following manufacturer’s instructions. Urine samples were processed directly according to the kit manual. For GAG in tissues quantification, individual pieces (approximately 50 mg) were removed from the frozen samples (liver and kidney), weighed and digested in 1 mL papain solution prepared according to the Blyscan kit protocol. After the extraction, 20 μL from each reaction were used for sulphated GAG determination following the Blyscan kit protocol. The obtained values for sulphated GAG were adjusted to the volume of papain reaction and were normalized to the weight of the tissue used or, in the case of urine samples, to creatinine levels.

#### Histological analysis

Quantification of the accumulation of granules in leukocytes was done after May-Grunwald–Giemsa staining of blood smears and microscopic examination. For the staining, premade solutions from Sigma Aldrich (May-Grunwald cat. N.: 63590 and Giemsa cat. N.: 48900) were used. Large leukocytes were assigned into three groups: 1) cells with no granules (no visible granules); 2) cells with low accumulation (containing between 1 and 10 granules); and, 3) cells with high level of accumulation (more than 10 granules). Only blood smears from WT chow diet, *Arsb*^*-*^ chow diet and *Arsb*^*-*^ treated with high dose of odiparcil (4.5 g/kg diet) were analyzed. Eosinophils were not included in the total number of leukocytes, since they were not assigned to any group 1), 2) or 3).

For histological assessment of cartilage thickness in knee and trachea, after paraffin embedding, 5 μm sections from knee and trachea were cut. The sections from the trachea were stained with Alcian Blue (0.1% in 3% Acetic Acid, pH 2.5, for 45 min then rinsed with water for 5 min). Under these conditions, Alcian Blue stains all proteoglycans which allows the visualization of cartilage layer and to measure its thickness. The thickness of individual tracheas was averaged from 10 measurements. The sections from the knee were stained with Fast Green and Safranin-O (0.03% Fast Green for 5 min, rinsed with 1% Acetic Acid for 15 s, 0.1% Safranin-O at pH 5.3 for 5 min, then rinsed with water for 5 min). This staining procedure allows the visualisation of cartilage growth plate and to measure the thickness of the distal femoral growth plate. The thickness of individual femoral growth plates was averaged from 5 measurements.

GAG tissue accumulation in liver and kidney sections was estimated based on Alcian Blue staining performed at low pH (pH 1), at which the dye stained predominantly sulphated GAG. Two slides at different levels per tissue block were used. Staining with 1% Alcian Blue in hydrochloric acid was performed on automated Leica Autostainer. After processing, the sections were imaged on a Laborlux D (Leitz) (100x, Zeiss ICc1 CCD camera with AxioVision software, 4 images by level were made; total 8 images by tissue). Image analysis was carried out using Visilog software. Images were corrected for illumination and pixels were sorted according to Red, Green and Blue intensities. Pixels from each image were sorted using the following algorithm: 1) which had first Red/Green intensities strictly inferior to 0.93 and Red/(Red + Green + Blue) strictly inferior to 0.326 and Blue/(Red + Green + Blue) strictly superior to 0.329, and 2) from these pixels, those that had Red/Green intensities between 0.89 and 0.93, and Red/(Red + Green + Blue) strictly superior to 199. Intensity of pixels that were rejected was put to 0. A proof image of these selected pixels was created for every input image. Then for each proof image, an RGB index was calculated that was the mean of Red + Green + Blue intensities of all non-black pixels. This RGB index was then reversed according to (-0.82608696 * RGB index) + 546.956522 (a low final index meant light Blue and a high final index meant intense Blue) and final index named BA index was calculated. In parallel, also the area of the non-black pixels in the proof image was calculated. To avoid artefacts due to differences in tissue preservation values for intensity were multiplied by stained area (“Area*Index” parameter). This calculated parameter gave the evaluation for the total signal of Alcian Blue staining for each image.

### Statistical analysis

For statistical analyses, commercial software (GraphPad Prism 5) was used. EC_50_ were calculated using the build in function Nonlinear fit for “log (inhibitor) vs. response—Variable slope (four parameters)”. For data from animal experiments Prism 5 1way ANOVA was used with Dunnet’s comparison to the untreated controls.

## Results

### Stimulation of secretion of sulphated CSGAG *in vitro* in bovine aortic endothelial cells (BAE) by odiparcil treatment

**Fig 1:**
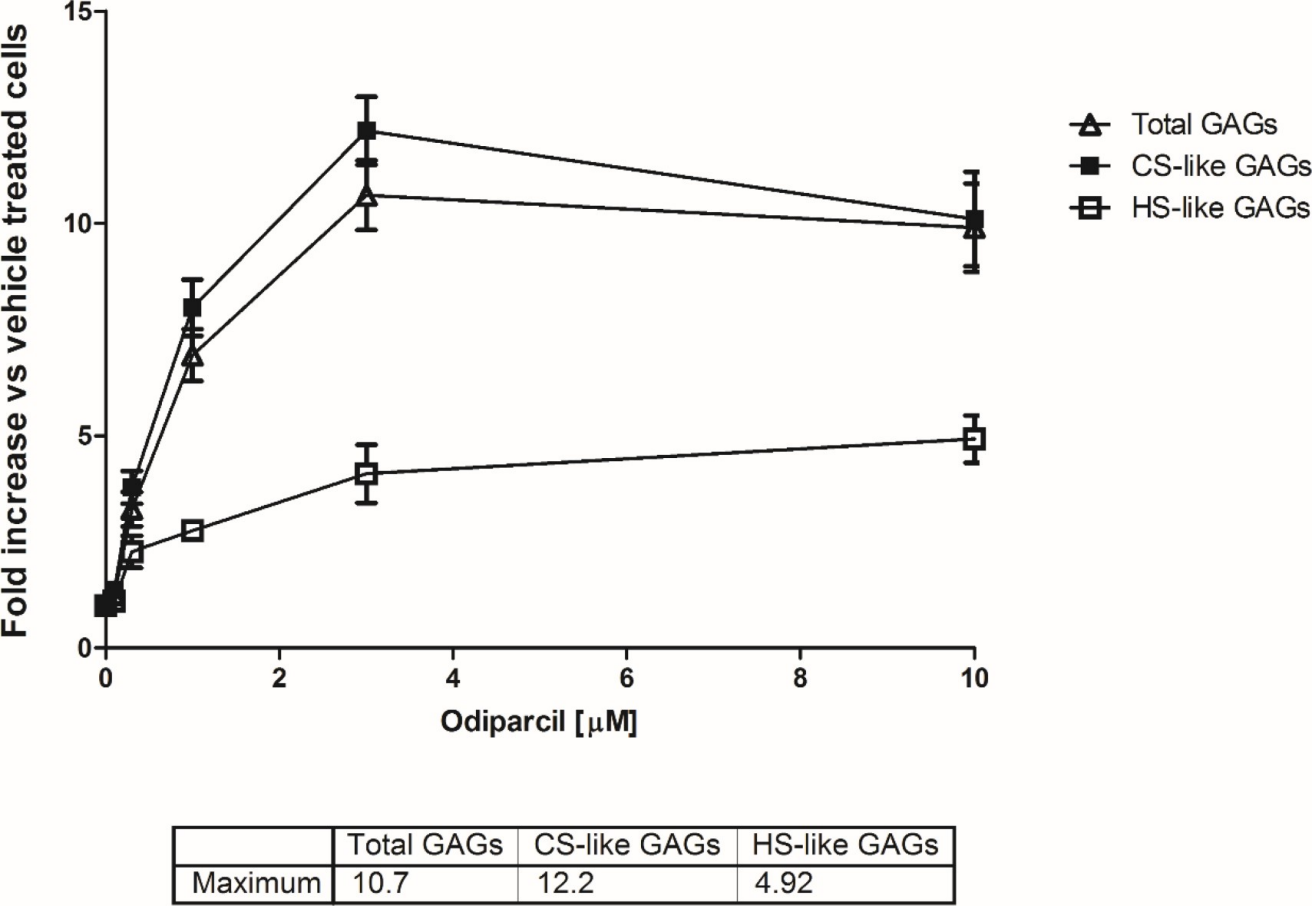
Stimulation by odiparcil of GAG secretion in BAE cells. Fold stimulation was calculated as folds over the base level (= 1). The table below the graph shows the maximum level of stimulation achieved for sulphated GAG, CSGAG and HSGAG.

To demonstrate odiparcil capability to stimulate cellular secretion of sulphated GAG species, we treated BAE cells with different concentrations of odiparcil and measured secretion of sulphated GAG in culture medium by metabolic labelling with [^35^S] as total sulphated GAG (containing CS/DS and HS) (Fig 1). Odiparcil, at micromolar concentrations dose dependently increased the secretion of total sulphated GAG (Fig 1). Maximum stimulation of total GAG secretion was achieved with 3 μM of odiparcil (10.7 fold over the base level). GAG types analysis of the newly synthesized sulphated GAG showed that they were mainly of CS type (CS type of GAG comprises CS and DS) (at 10 μM odiparcil, on average 73.5% from total detected sulphated GAG, S1 Table). In addition, HSGAG (Heparan sulphate GAG) were detected, but at lower levels (at 10 μM odiparcil, on average 19.5% from the total sulphated GAG, S1 Table) with maximum stimulation of HSGAG secretion at 10 μM odiparcil (4.9 fold over the base level).

### Chondroitin sulphate cellular distribution in skin fibroblasts from MPS VI donors

**Fig 2:**
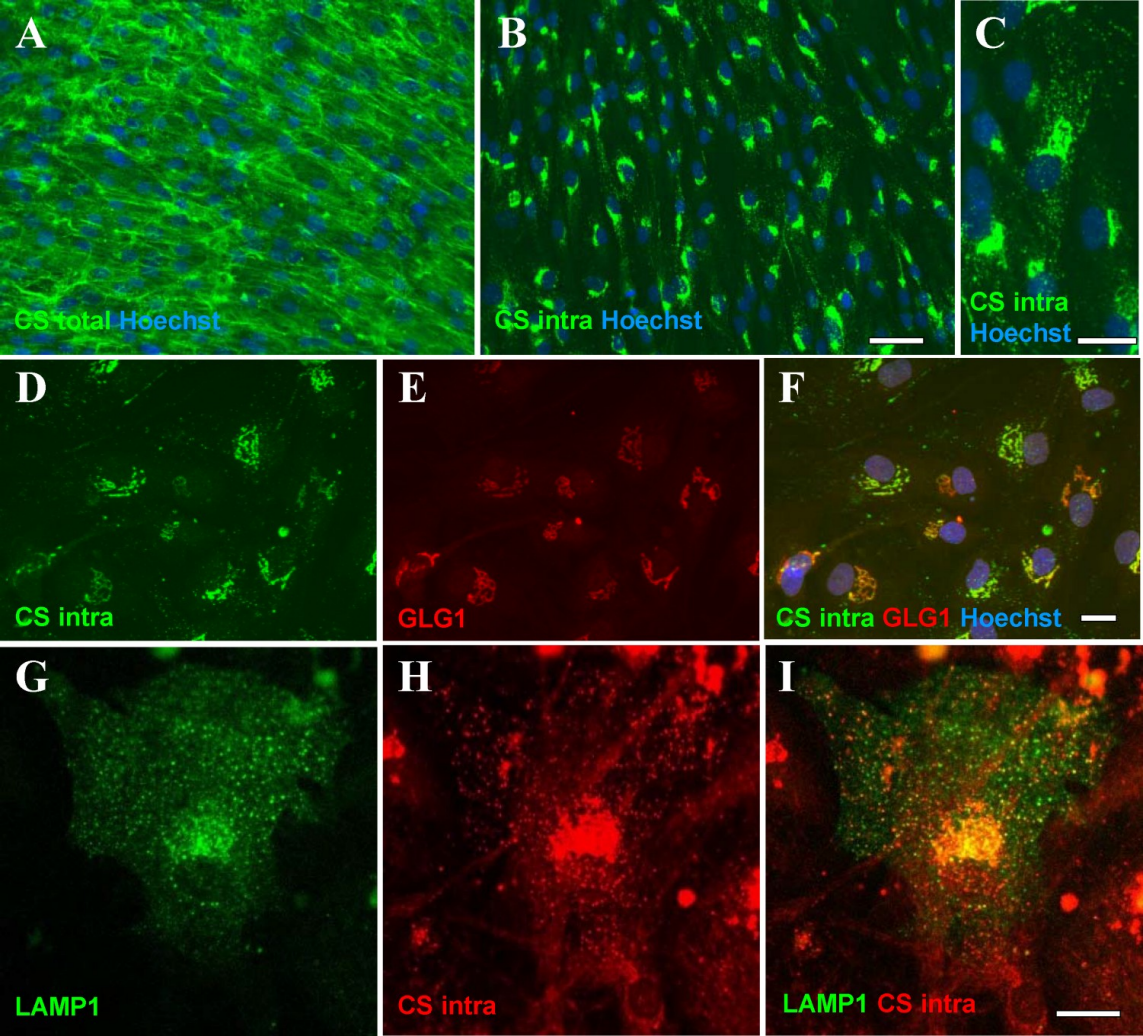
CS immunodetection and subcellular distribution in fibroblasts from MPS VI patient (GM00538). A, B [CS-56] detection of total cellular (A) and intracellular (B) CS in fibroblasts from MPS VI patient, C. high magnification of B. D, E and F. Partial co-localization of intracellular CS (D, imaging conditions chosen for best display of perinuclear CS staining) with GLG1 (Golgi) marker (E) seen on the overlay (F). G, H and I. Partial co-localization of intracellular CS (G, imaging conditions chosen for best display of dotty cytoplasmic CS staining) with LAMP1 (lysosomes) marker (H) seen on the overlay (I). Scale bars: A, B– 50 μm, C-I– 20 μm.

CSGAG (CS and DS) are the GAG species for which degradation is altered in MPS VI. On cellular level CS can be visualized by immunofluorescence with a CS specific antibody [CS-56] [32], however no such antibody was available for DS. Staining with [CS-56] antibody of human skin fibroblasts derived from MPS VI patients provided a strong uniform signal, indicating a high abundance of CS distributed in cells and on the cell’s surface (Fig 2A). Further, we could unmask the intracellular pool of CS by applying a protocol allowing predominantly staining of intracellular CS, in which prior to permeabilization the fibroblasts were treated with Chondroitinase ABC, digesting the CS attached to proteins either secreted into extracellular matrix or exposed on the cells surface. This protocol eliminated the predominant diffuse staining of total CS and revealed dotty patterns of intracellular CS positive structures (Fig 2B and 2C). Close to the nuclei these dotty structures appeared clustered, and sometimes mesh-like patterns were observed (Fig 2C and 2F). Since CS is mainly synthesized in Golgi apparatus [33–35], we performed a co-localization with anti-GLG1 (Golgi Glycoprotein 1) (Fig 2D, 2E and 2F) and found that the perinuclear CS positive structures co-localized, indicating the CS pool in the Golgi biosynthetic pathway. CS positive structures also partially co-localized with the lysosomal marker LAMP1-GFP (Fig 2G, 2H and 2I) confirming that some of the cytosolic CS positive structures represent lysosomes which was in accordance with MPS VI disease affecting lysosomal degradation of CS.

### Odiparcil mediated reduction of intracellular chondroitin sulphate levels in fibroblasts from MPS VI donors

**Fig 3:**
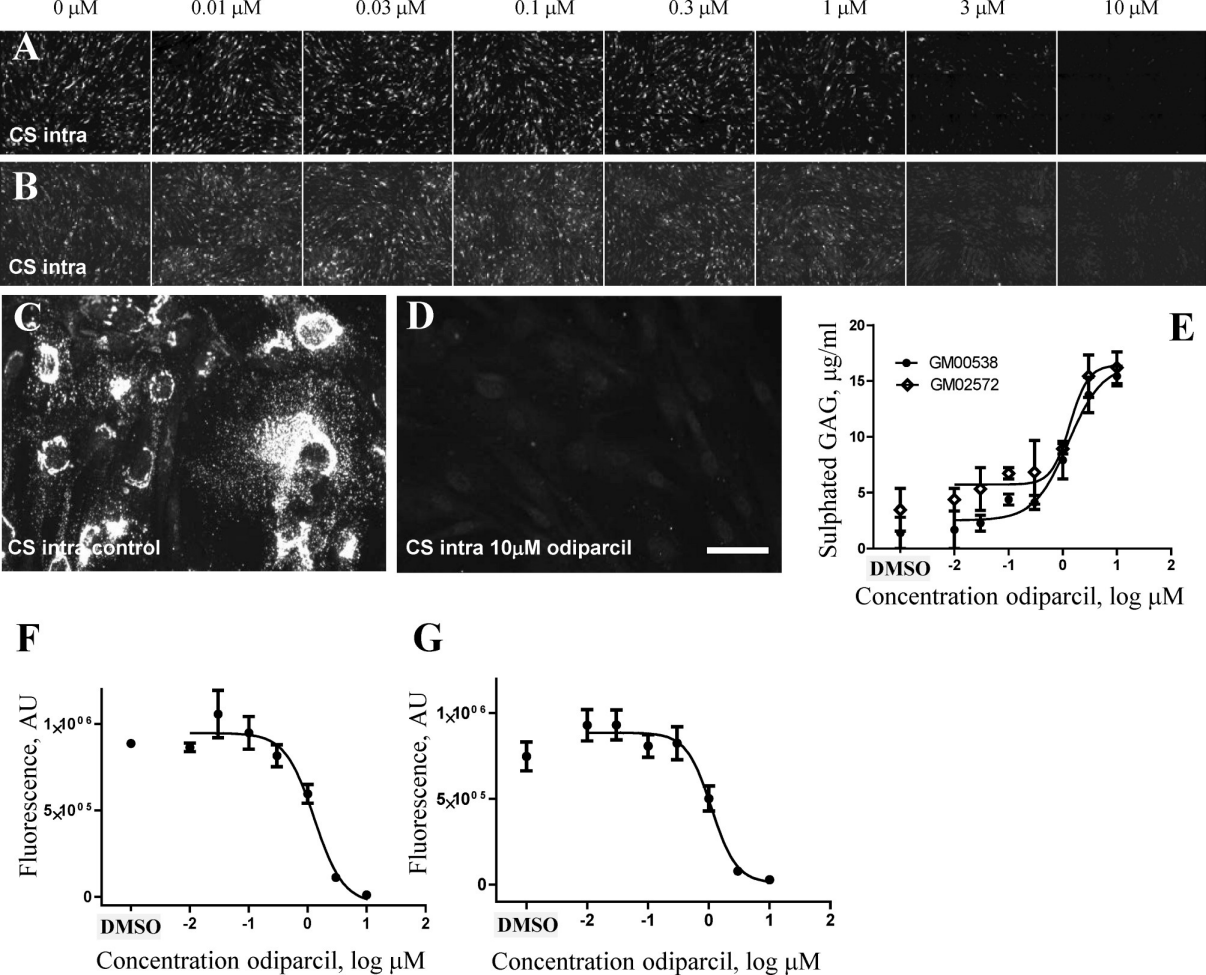
Odiparcil efficiently reduces the intracellular pool of CS in fibroblasts from MPS VI patients. A, B. Effect of odiparcil treatment on intracellular CS in MPS VI patient fibroblasts GM00538 in growing cells culture conditions (A) and in confluent cells culture conditions (B). C, D. High magnification of intracellular CS staining in MPS VI patient fibroblasts GM00538, no odiparcil treatment (C) and 10 μM odiparcil (D). E. Stimulation of excretion of sulphated GAG in cell culture medium by odiparcil treatment in MPS VI donors GM00538 and GM02572. F, G. Quantification of the effect of odiparcil on the intracellular CS in MPS VI patient fibroblasts GM00538 in growing cells culture conditions (F) and in confluent cells culture conditions (G). Scale bar: C, D– 50 μm. E-G—Data represented as mean ± SEM.

The effect of odiparcil on intracellular CS detected with [CS-56] antibody as described above was assessed *in vitro* in primary skin fibroblasts from donors with MPS VI disease (Fig 3 and S1 Fig). Odiparcil effectively reduced the intracellular pool of CS. The effect of odiparcil was measured in growing cells (odiparcil applied before cells reaching confluence) and in fibroblasts after reaching confluence (odiparcil applied 1 week after the cells reached confluence). In both systems, odiparcil decreased the intracellular pool of CS (Fig 3A–3D, 3F and 3G and S1 Fig). At 10 μM as seen at high magnification (Fig 3C and 3D and S1C and S1D Fig), odiparcil almost completely eliminated intracellular pool of CS. Similar depletion was seen in perinuclear and cytosolic CS positive structures implying that odiparcil treatment was effectively reducing CS in biosynthetic as well as in lysosomal structures. In addition, no effect on viability of the cells with odiparcil treatment was detected, as demonstrated by the similar number of cells at different odiparcil concentrations (S2 Fig). The calculated EC_50_ values for the reduction of CS levels ranged between 1 and 2 μM (Fig 3F and 3G, S1E and S1F Fig). Odiparcil increased total secreted sulphated GAG measured in the culture media (Fig 3E) with a similar EC_50_ value.

### Odiparcil tissue distribution

Odiparcil tissue distribution was studied in rat organs relevant for MPS VI disease (bone, cartilage, cornea and heart), and the corresponding tissue/plasma ratio (Kp) was calculated on AUC and corrected by the blood content in each organ. 30 min after last administration, odiparcil concentration in plasma was found to be ~ 7300 ng/mL (22.5 μM) (Table 1). All tested tissues were exposed to odiparcil after repeated dosing with the heart being the most exposed (Cmax ~4600 ng/g tissue (14.3 μM), AUC Kp ~0.44), followed by bone, cartilage and cornea (Cmax ~500 to 1300 ng/g (1.7 μM to 4 μM, AUC Kp ~0.15). Thus, repeated dosing with odiparcil led to Cmax in tested tissues above the EC_50_ measured for the reduction of intracellular CS when skin fibroblasts from MPS VI patients were treated with odiparcil.

**Table 1:** Odiparcil exposure in organs and plasma.

Matrix	Cmax (ng/mL or ng/g)	Cmax (μM)	AUC (ng.h/mL or ng.h/g)	Kp AUC
Plasma	7 297	22.5	14 807	-
Heart	4 628	14.3	10 203	0.69 (0.44^a^)
Bone	1 306	4.0	2 646	0.18
Cartilage	1 096	3.4	1 805	0.12
Cornea	535	1.7	1 737	0.12

^a^ blood corrected Kp AUC

### Monitoring progression of MPS VI disease by the increase of sulphated GAG in *Arsb*^*-*^ mice

**Fig 4:**
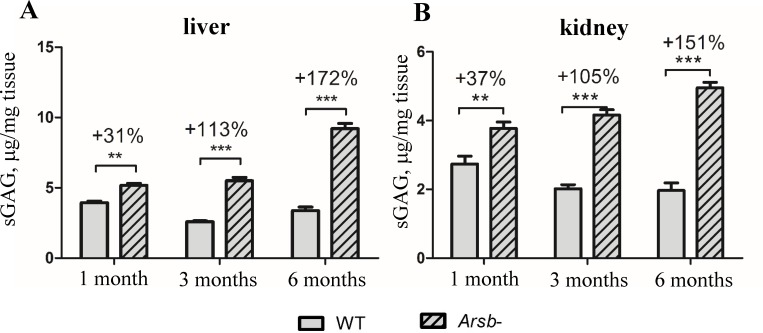
Progression of accumulation of GAG in organs of *Arsb*^*-*^ mice. Total GAG accumulation at 1, 3 and 6 months of age in liver (A) and kidney (B). Above bars for *Arsb*^*-*^ shown percent increase in total GAG compared to WT siblings at the same age. Data represented as mean ± SEM;**: *p* value < 0.01; ***: *p* value < 0.001.

In MPS VI mouse models [30,36,37], consistent with the MPS VI disease physiopathology, an accumulation of total sulphated GAG can be demonstrated in liver and kidney either by Alcian Blue staining, specific for sulphated GAG (S3 Fig), or by measuring total GAG content (Fig 4). The progression of MPS VI phenotype in *Arsb*^*-*^ mice could be monitored by measuring the accumulation of sulphated GAG in liver and kidney at different time points (1, 3 and 6 months of age) (Fig 4). Already at 1 month of age the difference between WT and *Arsb*^*-*^ was detectable (31% higher GAG content for liver and 37% higher for kidney in *Arsb*^*-*^ compared to WT), at 3 months the difference was more pronounced (113% higher for liver and 105% for kidney) and for 6 months old mice the increase reached 172% and 151% for total GAG in *Arsb*^*-*^ mice liver and kidney, respectively. These data were used to set up studies of the effects of odiparcil in *Arsb*^*-*^ mice using two paradigms: an early disease protocol (treatment starting in young 1 month old animals) and an established disease protocol (treatment starting in older, 3 months old animals).

### Odiparcil stimulates excretion of urinary sulphated GAG in *Arsb*^*-*^ mice

**Fig 5:**
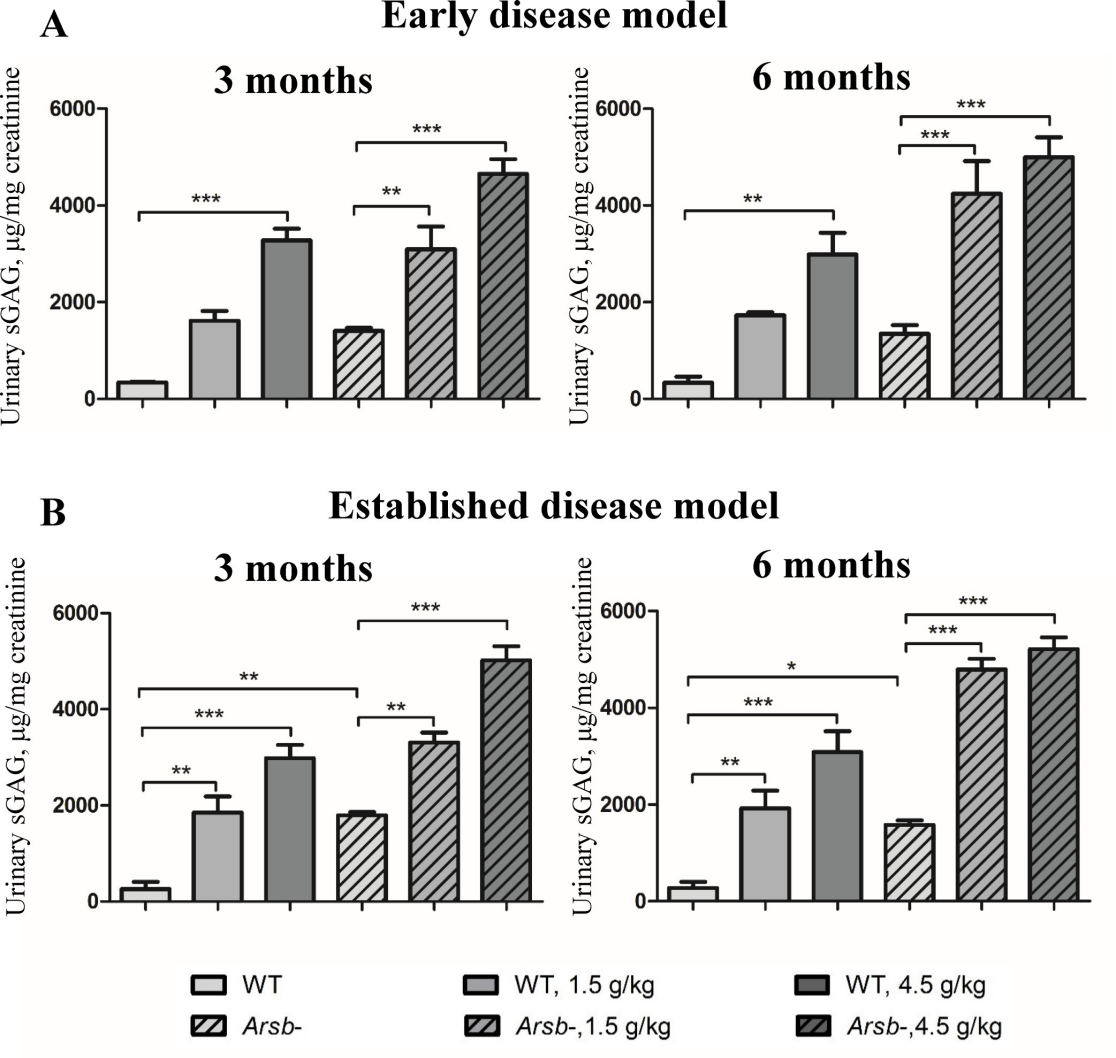
Stimulation of GAG excretion in urine of mice treated with odiparcil. Stimulation of GAG excretion after 3 and 6 months of treatment in early disease model (A) and in advanced disease model (B). Data represented as mean ± SEM;*: p-value<0.05, **: *p* value < 0.01; ***: *p* value < 0.001.

During studies in animals receiving odiparcil orally for more than 6 months, the effect of odiparcil treatment was measured at different time points using the excreted total sulphated GAG in urine (Fig 5). Insufficient cellular degradation of GAG in MPS diseases is detected by elevated GAG in urine. Accordingly, control *Arsb*^*-*^ mice showed elevated levels of urinary sulphated GAG (Fig 5). As expected, odiparcil treatment causing clearance of cellular GAG by diverting synthesis of GAG into soluble secretable odiparcil-bound GAG species led to an elevation of total GAG in urine (Fig 5). This elevation was detected in both wild type and *Arsb*^*-*^ mice, in the *Arsb*^*-*^ mice being above the pathology induced basal increase. Thus, elevation of urinary GAG in *Arsb*^*-*^ mice treated with odiparcil might be considered as a sum of excreted GAG induced by MPS pathology and odiparcil-bound GAG. This effect of odiparcil treatment on urinary GAG in both wild type and *Arsb*^*-*^ mice was dose-dependent and similar in both early and advanced disease protocols. The urinary GAG effect was consistent throughout the treatment period since no difference was observed at the two time points (after 3 months and 6 months treatment). In addition, odiparcil induced GAG were efficiently eliminated from the body, since two weeks after discontinuation of the treatment no elevated GAG were detected on top of the basal level in *Arsb*^*-*^ mice (S4 Fig).

### Odiparcil treatment reduces liver and kidney sulphated GAG accumulation in *Arsb*^*-*^ mice

**Fig 6:**
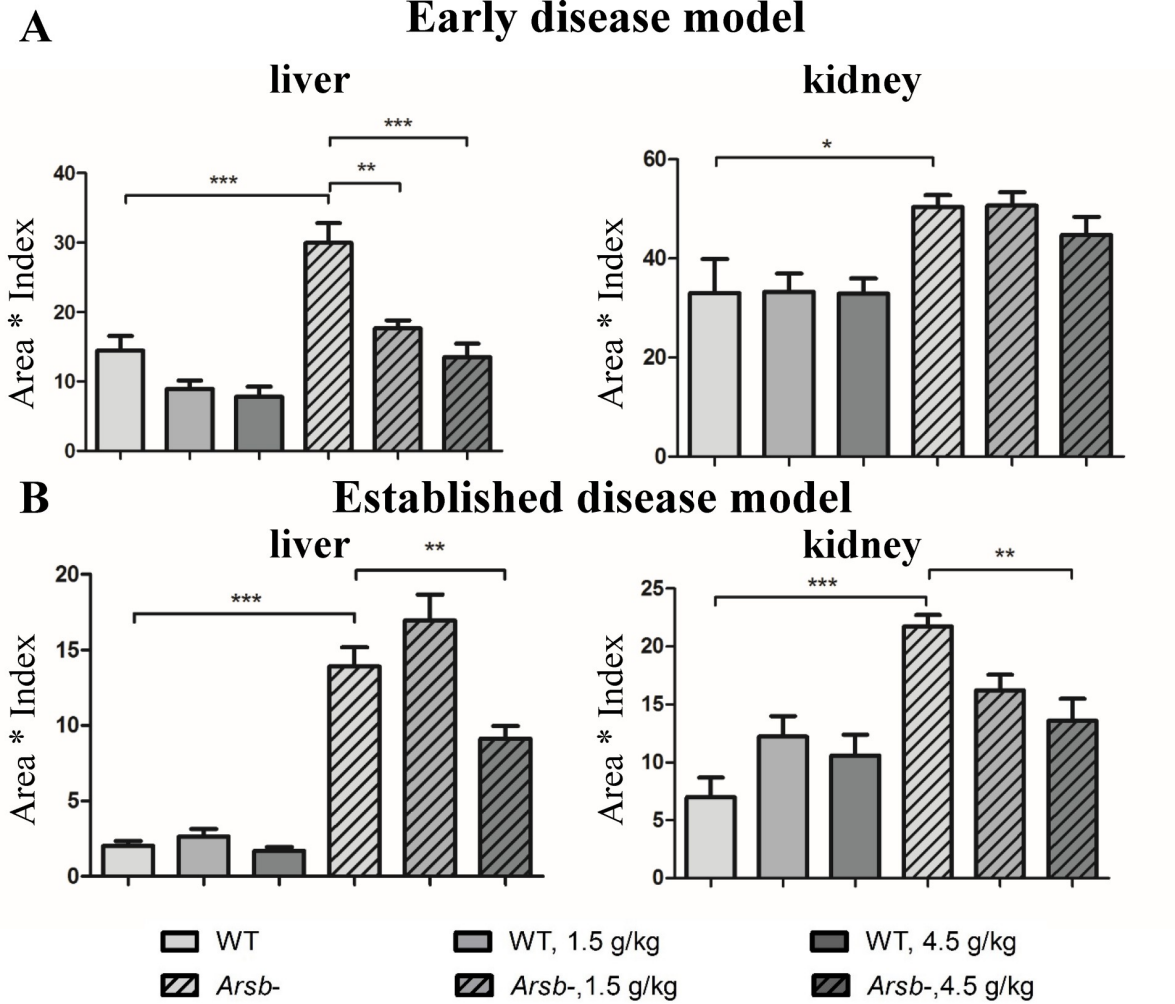
Odiparcil efficiently reduces the accumulation of sulphated GAG in liver and kidney in *Arsb*^*-*^ mice. Effect of odiparcil treatment on Alcian Blue staining in liver and kidney of *Arsb*^*-*^ mice in the early disease model (A) and in the advanced disease model (B). Data represented as mean ± SEM;*: p-value<0.05, **: *p* value < 0.01; ***: *p* value < 0.001.

The effect of odiparcil after 6 months of treatment on the accumulation of sulphated GAG in tissues of *Arsb*^*-*^ mice was assessed by evaluation of total GAG levels relative to WT in liver and kidney by Alcian Blue staining (Fig 6) or by measurement of total GAG by the Blyscan method (S5 Fig). In liver and kidney samples from control *Arsb*^*-*^ mice sulphated GAG were elevated consistently with the MPS VI disease phenotype (non-treated *Arsb*^*-*^ mice). Odiparcil significantly reduced liver GAG at both doses in the early disease model and at the highest dose in the established disease model. The effect of GAG reduction detected with Alcian Blue was more prominent, probably due to the staining more selectively detecting the excess GAG accumulating in the *Arsb*^*-*^ mice than the Blyscan method. Odiparcil also reduced kidney GAG, however the detected effect with Alcian Blue was significant only in the latter model. Notably, no significant effects on GAG levels in wild type animals treated with the two doses of odiparcil were observed.

### Odiparcil treatment reduces cartilage thickness in trachea and femoral growth plate in *Arsb*^*-*^ mice

**Fig 7:**
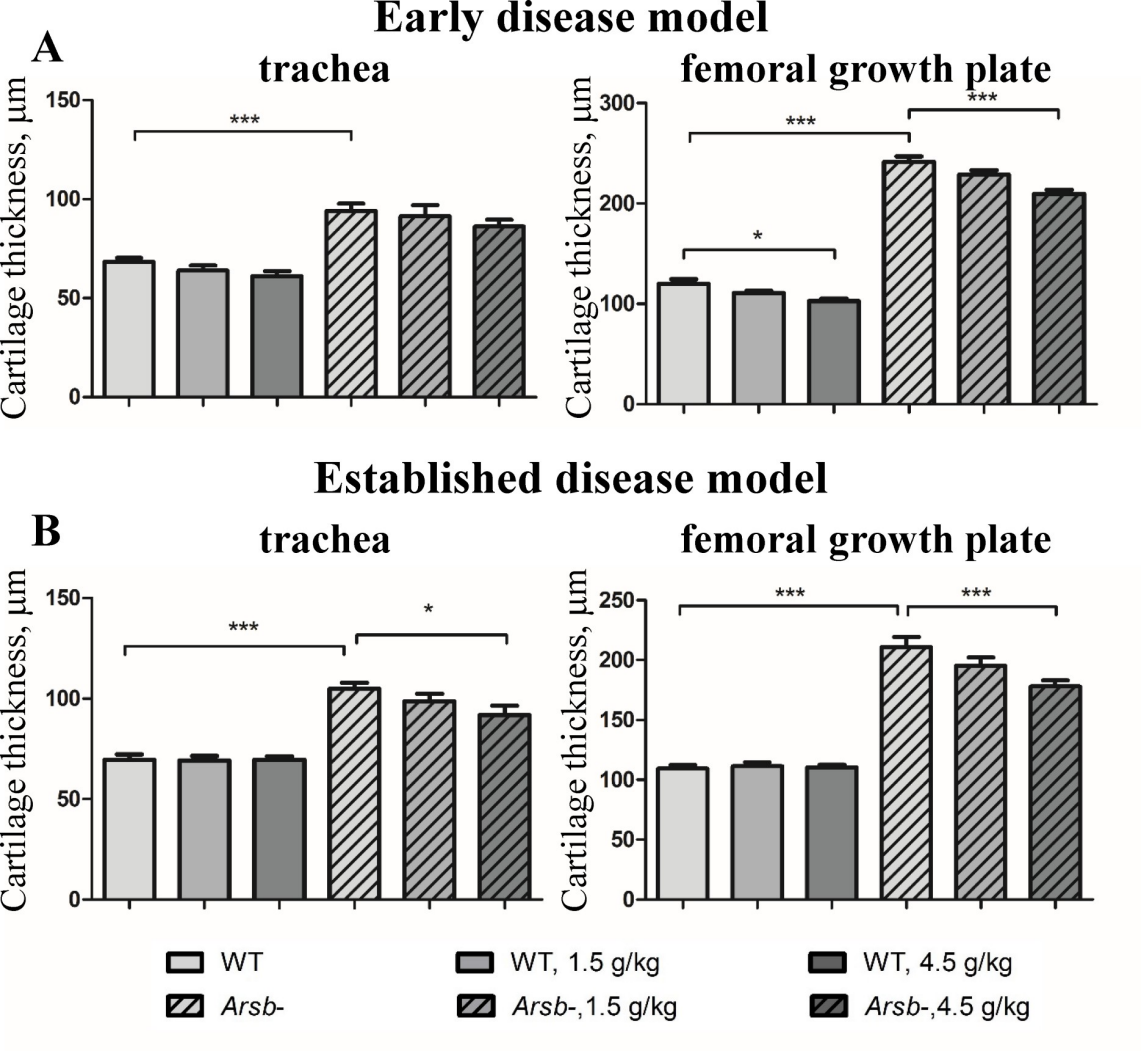
Odiparcil efficiently reduces thickening in cartilage of femoral growth plate and trachea in *Arsb*^*-*^ mice measured on histological sections. Dose dependency of odiparcil treatment on cartilage thickening in trachea and femoral growth plate of *Arsb*^*-*^ mice in the early disease model (A) and in the established disease model (B). Data represented as mean ± SEM;*: p-value<0.05, **: *p* value < 0.01; ***: *p* value < 0.001.

Increased thickness of cartilage in *Arsb*^*-*^ mice could be measured on histological sections from trachea and in the bones’ growth plates (distal femoral growth plate) (Fig 7, non-treated controls), consistent with the effects on cartilage in MPS VI disease [6,38]. In both early and established disease models after 6 months of treatment, a dose-dependent reduction of cartilage thickening was observed in odiparcil treated mutant animals (Fig 7). Some decrease in the thickness of cartilage was also observed for the wild type animals treated with odiparcil in the early disease treatment model (Fig 7A). This decrease was only observed in the distal femoral growth plate and it reached statistical significance only for the high dose treated animals (Fig 7A).

### Odiparcil treatment reduces accumulation of granules in leukocytes of *Arsb*^*-*^ mice

**Fig 8:**
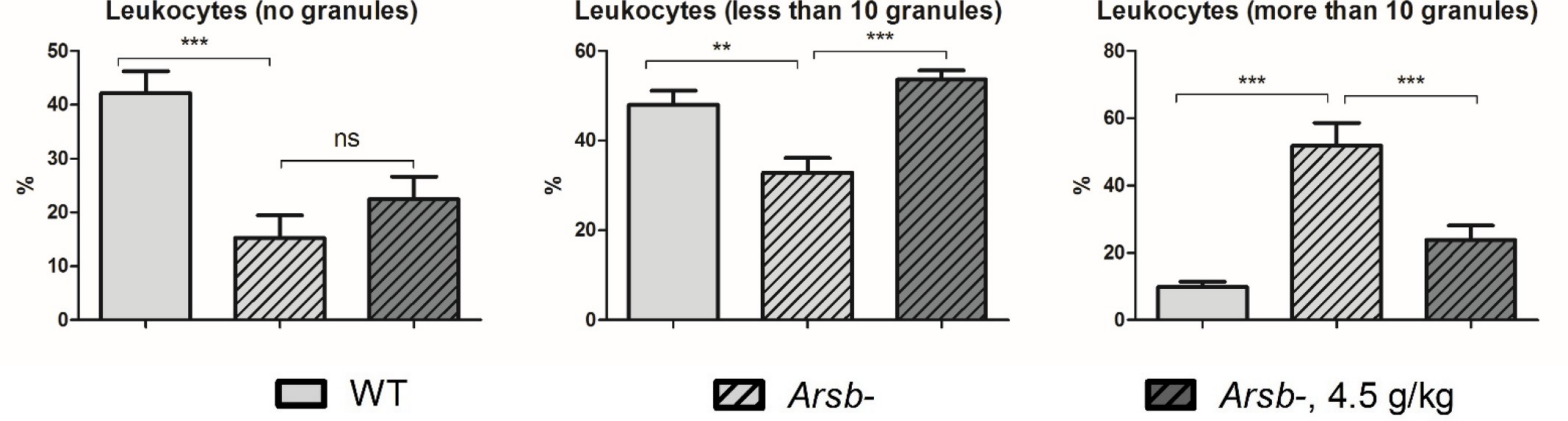
Odiparcil efficiently reduces number of leukocytes with high level of granules. Only blood smears from non-treated controls and high dose odiparcil treated *Arsb*^*-*^ were analysed. Data represented as mean ± SEM; ns: *p* value > 0.05, **:—*p* value < 0.01; ***: *p* value < 0.001.

A typical feature of MPS VI is the appearance of leukocytes with abnormal granules observed in both patients and animal models [39,40]. The appearance of these granules is consistent with high GAG accumulation in MPS VI leukocytes and the prominence of granules could be used to estimate the effect of odiparcil on intracellular GAG accumulation. Consistent with the MPS VI model, blood smears stained with May-Grunwald-Giemsa stains from WT and *Arsb*^*-*^ mice showed a difference in the number of visible granules (Fig 8, non-treated controls) with *Arsb*^*-*^ animals showing increase in leukocytes with high granules accumulation. In contrast, blood smears from early disease treatment studies in *Arsb*^*-*^ animals treated for 6 months with odiparcil at high dose, revealed reduced number of leukocytes with high granules accumulation (Fig 8). This is consistent with less GAG accumulating in fewer intracellular granules in *Arsb*^*-*^ treated animals.

## Discussion

The aim of this study was to evaluate the potential of odiparcil as an orally available GAG clearance therapy for MPS VI. Currently, the main therapy for MPS VI is ERT which is a weekly infusion of galsulfase. To reduce the pathological GAG content increase in MPS VI disease, we suggest an alternative approach where the levels of intracellular GAG are reduced by odiparcil, a β-D-xyloside analogue, substrate for β4GalT7. β-D-xylosides have been used to manipulate synthesis of proteoglycans and their ability to divert GAG synthesis has been investigated in thrombosis [18,25,41,42], cancer models [43,44] and in tissue regeneration [45–47]. Our data suggest that odiparcil can be effectively used in MPS VI therapy. The therapeutic potential of odiparcil in MPS VI is supported by the ability of the compound: *i*) to divert synthesis of cellular GAG into secretable GAG in BAE cells; *ii*) to reduce intracellular CS (as representative of GAG species with affected metabolism in MPS VI) while stimulating the secretion of soluble GAG in skin fibroblasts from MPS VI patients; *iii*) *in vivo*, in MPS VI mice, to reduce the pathological GAG accumulation in liver and kidney, to reduce granule accumulation in leukocytes and also to reduce the cartilage thickening observed in trachea and femoral growth plate. In addition, odiparcil therapy appears to be efficient irrespective of the disease stage since its therapeutic efficacy *in vivo* was similar when treatment was initiated in juvenile (aged up to 4 weeks) or in adult (aged 3 months) MPS VI mice, as well as *in vitro* in treating intracellular CS in dividing fibroblasts and in non–dividing confluent fibroblasts.

Odiparcil therapeutic approach in MPS VI is based on the drug ability to serve as a substrate for β4GalT7 thus redirecting GAG synthesis into secretable odiparcil-bound GAG which enter circulation and are readily eliminated via urine. This notion is supported by the fact that additional GAG were detected only in urine of odiparcil treated animals and not in the tissues (e.g. liver, kidney). Interestingly, the reduction of tissue GAG was only seen in MPS VI animals and not in the odiparcil treated wild type animals suggesting that the drug leads specifically to clearance of the accumulated GAG in the MPS VI pathology. Urinary GAG levels have been used for the diagnosis of MPS and for the evaluation of the efficacy of ERT [48,49]. In contrast to ERT, odiparcil treatment leads to increase of urinary GAG, however these are odiparcil-induced and odiparcil-bound GAG which are easily excreted and do not accumulate in tissues. Thus, the increase in the level of urinary GAG after odiparcil treatment can be used as a pharmacodynamics marker.

In MPS pathology, GAG accumulate in various cells, tissues and organs [3,4,50]. As a proof of principle, the effect of odiparcil on GAG accumulation in MPS VI treated mice was shown in liver and kidney (by the reduction in Alcian Blue staining and total GAG) and in leukocytes (by the reduction in the proportion of leukocytes with high number of granules). The effect in leukocytes and the ease of leukocytes isolation from peripheral blood suggest that leukocyte GAG content could be used as a marker of clinical efficacy in MPS VI patients treated with odiparcil, similar to that which has been already investigated in leukocytes of MPS IVa patients treated with elosulfase alpha [51].

Despite the positive outcomes of ERT and HSCT in MPS VI patients, there is still a significant unmet medical need due to the failure of current therapies to target poorly vascularized or barrier protected tissues such as bone, connective tissue, cornea, retina, cardiac valves and respiratory tissues. This failure results in severe clinical manifestations mostly seen in adult patients. There are also practical issues around delivering ERT to patients as it is based on frequent intravenous infusions. Another disadvantage of ERT is the production of antibodies against the recombinant enzymes which may contribute to some patients not responding maximally [52,53]. These factors lead to patients, patient groups and clinicians exploring either new or adjuvant therapies that could address the unmet needs. An example of a tissue where odiparcil would be beneficial compared to the ERT is cartilage in which ERT has poor penetration and limited effect [54,55] but which was found to respond to odiparcil treatment as seen by the reduction in cartilage thickening in *Arsb*^*-*^ mice after odiparcil treatment. Cornea is another tissue which is poorly exposed to recombinant enzyme N-acetylgalactosamine-4-sulfatase but was found to be exposed to odiparcil after repeated dosing. However, how this exposure to odiparcil translates in the MPS VI corneal GAG accumulation or eye functionality remains to be investigated.

Therapy for MPS VI with a small molecule such as odiparcil would have several advantages over existing therapies. Oral administration of odiparcil would reduce the burden of the weekly intravenous infusion of ERT and therefore improve quality of life of patients. Odiparcil is distributed into tissues and organs such as bones, cartilage and cornea where recombinant enzymes have poor penetration [56]. Our data with dosing in rats also indicate that odiparcil has high rates of tissue uptake. Odiparcil, similar to other β-D-xylosides, should not be immunogenic in contrast to ERT, where antibodies against exogenous enzymes are often produced in patients [57]. Based on the fact that the levels of sulphated GAG in urine were similar throughout the treatment period, we can conclude that there is no adaptation to odiparcil treatment which would potentially lead to the diminishing effect of the treatment over time. It would be also interesting to assay the sustainability of GAG tissue reduction over time after the end of odiparcil treatment. The persistence of the odiparcil effect might be related to the rate of GAG tissue accumulation in MPS VI pathology.

Lysosomal accumulation of non-degraded and partially degraded GAG is recognized as a primary defect leading to physiopathology in MPS [3–5]. It has been broadly accepted that this accumulation leads to a pathogenic cascade of events causing complex clinical manifestations in different MPS [6]. Thus, pathologically lower degradation of GAG might lead not only to increase of the lysosomal GAG (and their derivatives) but also to changes in levels of cellular and extracellular proteoglycans. The proteoglycans homeostasis in MPS is affected as indicated by alteration of activity and expression of various matrix proteases (such as metalloproteinases) and cathepsin usually found in lysosomes [58–60]. The proteoglycans changes / misregulation in turn affect cellular and tissue homeostasis including various signalling processes such as inflammation (e.g. via TNF-alpha [61,62]) and cell growth and differentiation (such as TGF-beta [63]). Odiparcil therapy provides a mechanism to interfere with the level of proteoglycans and our data show that it is effective in altering the glycosaminoglycan part of the proteoglycans, however the exact effect on proteoglycans and their regulation as well as the consequences on proteoglycan functionality in the cells and tissues remains to be unravelled.

Another intriguing question remaining is whether the odiparcil-bound GAG synthesized by various tissues elicit biological functions in the organism. The xyloside primed GAG are different in structure [22,64,65] and might directly elicit biological functions (e.g. as shown for cancer cells death [44]). Until now, the only studied effect of odiparcil-bound GAG has been the *in vivo* antithrombotic effect of the DS type odiparcil-bound GAG secreted into vasculature [18]. However, these odiparcil studies were done in wild type animals and the antithrombotic effect in MPS VI might be different since in MPS differences have been observed in the heparin cofactor II [66] which is the target of the odiparcil initiated DS GAG [18,25]. *In vivo* evaluation of the effects of odiparcil-bound GAG in tissues might require also technically challenging methodological discrimination between odiparcil-bound GAG and endogenous GAG. Extra level of complexity might be added by tissue and cell type specificity since the cell type influences the structure of xylosides-bound GAG in terms of CS/DS and HS proportion and disaccharide composition [44,65,67]. It has been also established that xylosides lead to changes in structure and functional properties of endogenous GAG [22,26,64]. Thus, to fully characterize the effects of odiparcil it will be necessary to analyse odiparcil-bound GAG, their effects and relationship with the endogenous GAG in various tissues, and also particularly, in relation to the MPS pathology.

The effects detected on the odiparcil-mediated production of GAG appear to be mainly on the CS type of GAG (comprising CS and DS) as seen *in vitro* by the analysis of the types of secreted GAG from BAE cells after odiparcil treatment. Previously, it has been demonstrated that odiparcil and its analogues engage production of circulating DS [18,25,41,42]. In addition, our fibroblast data demonstrate a direct effect on intracellular CS. Taken together, these data imply the suitability of odiparcil therapy primarily for MPS VI with altered CS and DS degradation either as a standalone therapy or an add-on to the existing therapies for MPS VI such as ERT. Some efficiency of odiparcil therapy for other MPS types with CS and/or DS accumulation (MPS I, II, IVa and VII) could be also suggested. In addition to the effects on CSGAG, the direct or indirect effects of odiparcil on other GAG types (HS and KS) need to be further investigated.

Previously, odiparcil has been developed as a unique antithrombotic agent [42] based on its mechanism of action resulting in the production of circulating GAG from which mainly DS can activate heparin cofactor II in turn inhibiting thrombin coagulation without bleeding liability. Furthermore, in more than 30 clinical trials that included a total of 1900 subjects, odiparcil has shown good safety and tolerability. These existing data on odiparcil safety could be used in the drug development for treatment of MPS VI.

## Conclusion

Our study unveils the potential of using odiparcil as a novel orally available drug for treatment of MPS VI. Based on the mechanism of action, by which odiparcil diverts synthesis of endogenous GAG into soluble secretable GAG, we show that *in vitro* in skin fibroblasts from MPS VI patients odiparcil reduces the intracellular CS pool and stimulates secretion of GAG into the culture medium. Odiparcil given orally was efficacious for treating *Arsb*^*-*^ mice, a model mimicking MPS VI disease. Odiparcil treatment led to a reduction of GAG accumulation in liver and kidney, reduction of cartilage thickening in trachea and femoral growth plate and reduction of granules accumulation in leukocytes. These results support the therapeutic potential of odiparcil in MPS diseases where CS and/or DS accumulate (such as MPS I, II, IVa, VI and VII).

## Supporting information

S1 Fig Odiparcil efficiently reduces the intracellular pool of CS in fibroblasts from MPS VI patient (data from donor GM02572). **A**, **B**. Effect of odiparcil treatment on intracellular CS in MPS VI patient fibroblasts GM02572 in growing cells culture conditions (A) and in confluent cells culture conditions (B). **C**, **D**. High magnification of intracellular CS staining in MPS VI patient fibroblasts GM02572, no odiparcil treatment (C) and 10 μM odiparcil (D). **E**, **F**. Quantification of the effect of odiparcil on the intracellular CS in MPS VI patient fibroblasts GM02572 in growing cells culture conditions (E) and in confluent cells culture conditions (F). Scale bar: C, D– 50 μm. E-G—Data represented as mean ± SEM.
(TIF)

S2 Fig Odiparcil treatment has no effect on number of cultured cells during treatment. Mean number of cells from MPS VI donor GM00538 per examination field after odiparcil treatment in growing cell culture conditions (A) and in confluent cell culture conditions (B).
(TIF)

S3 Fig Accumulation of GAG in organs of *Arsb*^*-*^ mice revealed by Alcian Blue staining. Liver (A) and kidney (B) sections stained with Alcian Blue from mice at 6 months of age, note higher levels of Alcian Blue staining in *Arsb*^*-*^ liver and kidney.
(TIF)

S4 Fig Return of the level of urinary sulphated GAG to the basal level in *Arsb*^*-*^ mice as detected after 2 weeks from the discontinuation of odiparcil treatment. Data represented as mean ± SEM; ***: *p* value < 0.001.
(TIF)

S5 Fig Odiparcil efficiently reduces the accumulation of total sulphated GAG in liver and kidney in *Arsb*^*-*^ mice. Effect of odiparcil treatment on total GAG detected by Blyscan method in liver and kidney of *Arsb*^*-*^ mice in the early disease model (A) and in the advanced disease model (B). Data represented as mean ± SEM;*: p-value<0.05, **: *p* value < 0.01; ***: *p* value < 0.001.
(TIF)

S1 Table Relative presence of CSGAG and HSGAG in cell culture media of BAE cells treated with odiparcil. Percentage of secreted CSGAG (comprising CS and DS) and HSGAG were calculated as % from total GAG in individual separate reactions of degradation by specific enzyme (CSase ABC or Heparitinase II). That is why the sum of Mean CSGAG (%) and Mean HSGAG (%) at a given odiparcil concentration is not 100%.
(DOCX)

S1 File The arrive guidelines checklist. (PDF)

## References

[1] O'Brien JF, Cantz M, Spranger J. Maroteaux-Lamy disease (mucopolysaccharidosis VI), subtype A: Deficiency of a N-acetylgalactosamine-4-sulfatase. Biochem Biophys Res Commun. 1974; 60: 1170–1177. 10.1016/0006-291x(74)90435-5 . 4215420

[2] Fluharty AL, Stevens RL, Sanders DL, Kihara H. Arylsulfatase B deficiency in Maroteaux-Lamy syndrome cultured fibroblasts. Biochem Biophys Res Commun. 1974; 59: 455–461. 10.1016/s0006-291x(74)80001-x . 4277366

[3] Neufeld EF, Muenzer J. The mucopolysaccharidoses. The Metabolic and Molecular Bases of Inherited Disease. New York: McGraw Hill: Scriver C, Beaudet A, Sly W et al; 2001 pp. 3421–3452.

[4] Valayannopoulos V, Nicely H, Harmatz P, Turbeville S. Mucopolysaccharidosis VI. Orphanet J Rare Dis. 2010; 5: 5 10.1186/1750-1172-5-5 . 20385007 PMC2873242

[5] Harmatz P, Shediac R. Mucopolysaccharidosis VI: pathophysiology, diagnosis and treatment. Front Biosci (Landmark Ed). 2017; 22: 385–406. 10.2741/4490 . 27814620

[6] Clarke LA. Pathogenesis of skeletal and connective tissue involvement in the mucopolysaccharidoses. Glycosaminoglycan storage is merely the instigator. Rheumatology (Oxford). 2011; 50 Suppl 5: v13–8. 10.1093/rheumatology/ker395 . 22210665

[7] Clarke LA, Hollak CEM. The clinical spectrum and pathophysiology of skeletal complications in lysosomal storage disorders. Best practice & Research clinical endocrinology & Metabolism. 2015; 29: 219–235. 10.1016/j.beem.2014.08.010 . 25987175

[8] Braunlin EA, Harmatz PR, Scarpa M, Furlanetto B, Kampmann C, Loehr JP, et al Cardiac disease in patients with mucopolysaccharidosis. Presentation, diagnosis and management. J Inherit Metab Dis. 2011; 34: 1183–1197. 10.1007/s10545-011-9359-8 . 21744090 PMC3228957

[9] Golda A, Jurecka A, Tylki-Szymanska A. Cardiovascular manifestations of mucopolysaccharidosis type VI (Maroteaux-Lamy syndrome). Int J Cardiol. 2012; 158: 6–11. 10.1016/j.ijcard.2011.06.097 . 21737154

[10] Fong LV, Menahem S, Wraith JE, Chow CW. Endocardial fibroelastosis in mucopolysaccharidosis type VI. Clin Cardiol. 1987; 10: 362–364. 10.1002/clc.4960100612 . 3109796

[11] Ashworth JL, Biswas S, Wraith E, Lloyd IC. The ocular features of the mucopolysaccharidoses. Eye (Lond). 2006; 20: 553–563. 10.1038/sj.eye.6701921 . 15905869

[12] Cantor LB, Disseler JA, Wilson FM. Glaucoma in the Maroteaux-Lamy syndrome. Am J Ophthalmol. 1989; 108: 426–430. 10.1016/s0002-9394(14)73311-2 . 2508477

[13] Giugliani R, Federhen A, Vairo F, Vanzella C, Pasqualim G, da Silva LMR, et al Emerging drugs for the treatment of mucopolysaccharidoses. Expert Opin Emerg Drugs. 2016; 21: 9–26. 10.1517/14728214.2016.1123690 . 26751109

[14] Sawamoto K, Stapleton M, Alméciga-Díaz CJ, Espejo-Mojica AJ, Losada JC, Suarez DA, et al Therapeutic Options for Mucopolysaccharidoses: Current and Emerging Treatments. Drugs. 2019; 79: 1103–1134. 10.1007/s40265-019-01147-4 . 31209777

[15] Harmatz P, Whitley CB, Waber L, Pais R, Steiner R, Plecko B, et al Enzyme replacement therapy in mucopolysaccharidosis VI (Maroteaux-Lamy syndrome). J Pediatr. 2004; 144: 574–580. 10.1016/j.jpeds.2004.03.018 . 15126989

[16] Brunelli MJ, Atallah ÁN, da Silva EMK. Enzyme replacement therapy with galsulfase for mucopolysaccharidosis type VI. Cochrane Database Syst Rev. 2016; 3: CD009806 10.1002/14651858.CD009806.pub2 . 26943923

[17] Fenzl CR, Teramoto K, Moshirfar M. Ocular manifestations and management recommendations of lysosomal storage disorders I: mucopolysaccharidoses. Clin Ophthalmol. 2015; 9: 1633–1644. 10.2147/OPTH.S78368 . 26379420 PMC4567234

[18] Toomey JR, Abboud MA, Valocik RE, Koster PF, Burns-Kurtis CL, Pillarisetti K, et al A comparison of the beta-D-xyloside, odiparcil, to warfarin in a rat model of venous thrombosis. J Thromb Haemost. 2006; 4: 1989–1996. 10.1111/j.1538-7836.2006.02064.x . 16961606

[19] Galligani L, Hopwood J, Schwartz NB, Dorfman A. Stimulation of synthesis of free chondroitin sulfate chains by beta-D-xylosides in cultured cells. J Biol Chem. 1975; 250: 5400–5406. . 167013

[20] Schwartz NB. Regulation of chondroitin sulfate synthesis. Effect of beta-xylosides on synthesis of chondroitin sulfate proteoglycan, chondroitin sulfate chains, and core protein. J Biol Chem. 1977; 252: 6316–6321. . 561070

[21] Lugemwa FN, Sarkar AK, Esko JD. Unusual beta-D-xylosides that prime glycosaminoglycans in animal cells. J Biol Chem. 1996; 271: 19159–19165. 10.1074/jbc.271.32.19159 . 8702593

[22] Chua JS, Kuberan B. Synthetic Xylosides. Probing the Glycosaminoglycan Biosynthetic Machinery for Biomedical Applications. Acc Chem Res. 2017; 50: 2693–2705. 10.1021/acs.accounts.7b00289 . 29058876

[23] Prydz K, Dalen KT. Synthesis and sorting of proteoglycans. J Cell Sci. 2000; 113 Pt 2: 193–205. . 10633071 10.1242/jcs.113.2.193

[24] Schwartz NB, Rodén L, Dorfman A. Biosynthesis of chondroitin sulfate: interaction between xylosyltransferase and galactosyltransferase. Biochem Biophys Res Commun. 1974; 56: 717–724. 10.1016/0006-291x(74)90664-0 . 4857056

[25] Masson PJ, Coup D, Millet J, Brown NL. The effect of the beta-D-xyloside naroparcil on circulating plasma glycosaminoglycans. An explanation for its known antithrombotic activity in the rabbit. J Biol Chem. 1995; 270: 2662–2668. 10.1074/jbc.270.6.2662 . 7852334

[26] Weinstein T, Evron Z, Trebicz-Geffen M, Aviv M, Robinson D, Kollander Y, et al β-D-xylosides stimulate GAG synthesis in chondrocyte cultures due to elevation of the extracellular GAG domains, accompanied by the depletion of the intra-pericellular GAG pools, with alterations in the GAG profiles. Connect Tissue Res. 2012; 53: 169–179. 10.3109/03008207.2011.620190 . 22149722

[27] Garud DR, Tran VM, Victor XV, Koketsu M, Kuberan B. Inhibition of heparan sulfate and chondroitin sulfate proteoglycan biosynthesis. J Biol Chem. 2008; 283: 28881–28887. 10.1074/jbc.M805939200 . 18708345 PMC2570865

[28] Shaukat I, Barré L, Venkatesan N, Li D, Jaquinet J-C, Fournel-Gigleux S, et al Targeting of Proteoglycan Synthesis Pathway: A New Strategy to Counteract Excessive Matrix Proteoglycan Deposition and Transforming Growth Factor-β1-Induced Fibrotic Phenotype in Lung Fibroblasts. PLoS ONE. 2016; 11: e0146499 10.1371/journal.pone.0146499 . 26751072 PMC4709117

[29] Curtain MM, Donahue LR. A mutation in the Arsb gene; a mouse model that resembles Maroteaux-Lamy syndrome; 2009 MGI Direct Data Submission. Available: http://www.informatics.jax.org/reference/J:149960.

[30] Pohl S, Angermann A, Jeschke A, Hendrickx G, Yorgan TA, Makrypidi-Fraune G, et al The Lysosomal Protein Arylsulfatase B Is a Key Enzyme Involved in Skeletal Turnover. J Bone Miner Res. 2018; 33: 2186–2201. 10.1002/jbmr.3563 . 30075049

[31] Coulson-Thomas VJ, Gesteira TF. Dimethylmethylene Blue Assay (DMMB). Bio-protocol. 2014; 4: e1236 10.21769/BioProtoc.1236

[32] Avnur Z, Geiger B. Immunocytochemical localization of native chondroitin-sulfate in tissues and cultured cells using specific monoclonal antibody. Cell. 1984; 38: 811–822. 10.1016/0092-8674(84)90276-9 . 6435883

[33] Geetha-Habib M, Campbell SC, Schwartz NB. Subcellular localization of the synthesis and glycosylation of chondroitin sulfate proteoglycan core protein. J Biol Chem. 1984; 259: 7300–7310. . 6725288

[34] Stanley P. Golgi glycosylation. Cold Spring Harb Perspect Biol. 2011; 3 10.1101/cshperspect.a005199 . 21441588 PMC3062213

[35] Mikami T, Kitagawa H. Biosynthesis and function of chondroitin sulfate. Biochim Biophys Acta. 2013; 1830: 4719–4733. 10.1016/j.bbagen.2013.06.006 . 23774590

[36] Evers M, Saftig P, Schmidt P, Hafner A, McLoghlin DB, Schmahl W, et al Targeted disruption of the arylsulfatase B gene results in mice resembling the phenotype of mucopolysaccharidosis VI. Proc Natl Acad Sci U S A. 1996; 93: 8214–8219. 10.1073/pnas.93.16.8214 . 8710849 PMC38649

[37] Strauch OF, Stypmann J, Reinheckel T, Martinez E, Haverkamp W, Peters C. Cardiac and ocular pathologies in a mouse model of mucopolysaccharidosis type VI. Pediatr Res. 2003; 54: 701–708. 10.1203/01.PDR.0000084085.65972.3F . 12904606

[38] Nuttall JD, Brumfield LK, Fazzalari NL, Hopwood JJ, Byers S. Histomorphometric analysis of the tibial growth plate in a feline model of mucopolysaccharidosis type VI. Calcif Tissue Int. 1999; 65: 47–52. 10.1007/s002239900656 . 10369733

[39] Alroy J, Freden GO, Goyal V, Raghavan SS, Schunk KL. Morphology of leukocytes from cats affected with alpha-mannosidosis and mucopolysaccharidosis VI (MPS VI). Vet Pathol. 1989; 26: 294–302. 10.1177/030098588902600402 . 2503918

[40] Anderson G, Smith VV, Malone M, Sebire NJ. Blood film examination for vacuolated lymphocytes in the diagnosis of metabolic disorders; retrospective experience of more than 2,500 cases from a single centre. J Clin Pathol. 2005; 58: 1305–1310. 10.1136/jcp.2005.027045 . 16311352 PMC1770783

[41] Masson P, Theveniaux J, Coup D, Grégoire T, Vaillot M, Dupouy D, et al Further studies on the mechanism for the antithrombotic effects of naroparcil, an orally active thioxyloside compound. Thromb Haemost. 1999; 81: 945–950. . 10404773

[42] Myers AL, Upreti VV, Khurana M, Eddington ND. Characterization of total plasma glycosaminoglycan levels in healthy volunteers following oral administration of a novel antithrombotic odiparcil with aspirin or enoxaparin. J Clin Pharmacol. 2008; 48: 1158–1170. 10.1177/0091270008323751 . 18757783

[43] Mani K, Belting M, Ellervik U, Falk N, Svensson G, Sandgren S, et al Tumor attenuation by 2(6-hydroxynaphthyl)-beta-D-xylopyranoside requires priming of heparan sulfate and nuclear targeting of the products. Glycobiology. 2004; 14: 387–397. 10.1093/glycob/cwh035 . 14718369

[44] Persson A, Tykesson E, Westergren-Thorsson G, Malmström A, Ellervik U, Mani K. Xyloside-primed Chondroitin Sulfate/Dermatan Sulfate from Breast Carcinoma Cells with a Defined Disaccharide Composition Has Cytotoxic Effects in Vitro. J Biol Chem. 2016; 291: 14871–14882. 10.1074/jbc.M116.716829 . 27226567 PMC4938203

[45] Chua JS, Tran VM, Kalita M, Quintero MV, Antelope O, Muruganandam G, et al A glycan-based approach to therapeutic angiogenesis. PLoS ONE. 2017; 12: e0182301 10.1371/journal.pone.0182301 . 28763512 PMC5538652

[46] Smith-Thomas LC, Stevens J, Fok-Seang J, Faissner A, Rogers JH, Fawcett JW. Increased axon regeneration in astrocytes grown in the presence of proteoglycan synthesis inhibitors. J Cell Sci. 1995; 108 (Pt 3): 1307–1315. . 7622613 10.1242/jcs.108.3.1307

[47] Muto J, Naidu NN, Yamasaki K, Pineau N, Breton L, Gallo RL. Exogenous addition of a C-xylopyranoside derivative stimulates keratinocyte dermatan sulfate synthesis and promotes migration. PLoS ONE. 2011; 6: e25480 10.1371/journal.pone.0025480 . 21998662 PMC3187761

[48] Chuang C-K, Lin H-Y, Wang T-J, Tsai C-C, Liu H-L, Lin S-P. A modified liquid chromatography/tandem mass spectrometry method for predominant disaccharide units of urinary glycosaminoglycans in patients with mucopolysaccharidoses. Orphanet J Rare Dis. 2014; 9: 135 10.1186/s13023-014-0135-3 . 25178307 PMC4164790

[49] Zhang H, Wood T, Young SP, Millington DS. A straightforward, quantitative ultra-performance liquid chromatography-tandem mass spectrometric method for heparan sulfate, dermatan sulfate and chondroitin sulfate in urine: an improved clinical screening test for the mucopolysaccharidoses. Mol Genet Metab. 2015; 114: 123–128. 10.1016/j.ymgme.2014.09.009 . 25458519

[50] Alroy J, Lyons JA. Lysosomal Storage Diseases. Journal of Inborn Errors of Metabolism and Screening. 2014; 2: 1–20. 10.1177/2326409813517663

[51] Baldo G, Poswar F, Federhen A, Bittar C, Gus R, Bender F, et al Enzyme Replacement Therapy With Elosulfase Alfa Decreases Storage of Glycosaminoglycan in White Blood Cells of Patients With Morquio A Syndrome. Journal of Inborn Errors of Metabolism and Screening. 2015; 3: 1–3. 10.1177/2326409814567741

[52] Brooks DA, King BM, Crawley AC, Byers S, Hopwood JJ. Enzyme replacement therapy in Mucopolysaccharidosis VI: evidence for immune responses and altered efficacy of treatment in animal models. Biochim Biophys Acta. 1997; 1361: 203–216. 10.1016/s0925-4439(97)00036-7 . 9300802

[53] Debiec H, Valayannopoulos V, Boyer O, Nöel L-H, Callard P, Sarda H, et al Allo-immune membranous nephropathy and recombinant aryl sulfatase replacement therapy: a need for tolerance induction therapy. J Am Soc Nephrol. 2014; 25: 675–680. 10.1681/ASN.2013030290 . 24262793 PMC3968491

[54] Crawley AC, Brooks DA, Muller VJ, Petersen BA, Isaac EL, Bielicki J, et al Enzyme replacement therapy in a feline model of Maroteaux-Lamy syndrome. J Clin Invest. 1996; 97: 1864–1873. 10.1172/JCI118617 . 8621770 PMC507255

[55] Hendrickx G, Danyukova T, Baranowsky A, Rolvien T, Angermann A, Schweizer M, et al Enzyme replacement therapy in mice lacking arylsulfatase B targets bone remodeling cells, but not chondrocytes. Hum Mol Genet. 2020 10.1093/hmg/ddaa006 . 31943020 PMC7104678

[56] Kakkis ED, Schuchman E, He X, Wan Q, Kania S, Wiemelt S, et al Enzyme replacement therapy in feline mucopolysaccharidosis I. Mol Genet Metab. 2001; 72: 199–208. 10.1006/mgme.2000.3140 . 11243725

[57] Kishnani PS, Dickson PI, Muldowney L, Lee JJ, Rosenberg A, Abichandani R, et al Immune response to enzyme replacement therapies in lysosomal storage diseases and the role of immune tolerance induction. Mol Genet Metab. 2016; 117: 66–83. 10.1016/j.ymgme.2015.11.001 . 26597321

[58] Simonaro CM, D'Angelo M, Haskins ME, Schuchman EH. Joint and bone disease in mucopolysaccharidoses VI and VII: identification of new therapeutic targets and biomarkers using animal models. Pediatr Res. 2005; 57: 701–707. 10.1203/01.PDR.0000156510.96253.5A . 15746260

[59] Wilson S, Hashamiyan S, Clarke L, Saftig P, Mort J, Dejica VM, et al Glycosaminoglycan-mediated loss of cathepsin K collagenolytic activity in MPS I contributes to osteoclast and growth plate abnormalities. Am J Pathol. 2009; 175: 2053–2062. 10.2353/ajpath.2009.090211 . 19834056 PMC2774069

[60] Baldo G, Tavares AMV, Gonzalez E, Poletto E, Mayer FQ, Matte UdS, et al Progressive heart disease in mucopolysaccharidosis type I mice may be mediated by increased cathepsin B activity. Cardiovasc Pathol. 2017; 27: 45–50. 10.1016/j.carpath.2017.01.001 . 28104572

[61] Simonaro CM, Ge Y, Eliyahu E, He X, Jepsen KJ, Schuchman EH. Involvement of the Toll-like receptor 4 pathway and use of TNF-alpha antagonists for treatment of the mucopolysaccharidoses. Proc Natl Acad Sci U S A. 2010; 107: 222–227. 10.1073/pnas.0912937107 . 20018674 PMC2806747

[62] Eliyahu E, Wolfson T, Ge Y, Jepsen KJ, Schuchman EH, Simonaro CM. Anti-TNF-alpha therapy enhances the effects of enzyme replacement therapy in rats with mucopolysaccharidosis type VI. PLoS ONE. 2011; 6: e22447 10.1371/journal.pone.0022447 . 21887218 PMC3159569

[63] Alliston T. Chondroitin sulfate and growth factor signaling in the skeleton. Possible links to MPS VI. J Pediatr Rehabil Med. 2010; 3: 129–138. 10.3233/PRM-2010-0117 . 20628554 PMC2901997

[64] Vassal-Stermann E, Duranton A, Black AF, Azadiguian G, Demaude J, Lortat-Jacob H, et al A New C-Xyloside induces modifications of GAG expression, structure and functional properties. PLoS ONE. 2012; 7: e47933 10.1371/journal.pone.0047933 . 23110134 PMC3482234

[65] Persson A, Ellervik U, Mani K. Fine-tuning the structure of glycosaminoglycans in living cells using xylosides. Glycobiology. 2018; 28: 499–511. 10.1093/glycob/cwy049 . 29800297

[66] Randall DR, Colobong KE, Hemmelgarn H, Sinclair GB, Hetty E, Thomas A, et al Heparin cofactor II-thrombin complex: a biomarker of MPS disease. Mol Genet Metab. 2008; 94: 456–461. 10.1016/j.ymgme.2008.05.001 . 18511319

[67] Persson A, Gomez Toledo A, Vorontsov E, Nasir W, Willén D, Noborn F, et al LC-MS/MS characterization of xyloside-primed glycosaminoglycans with cytotoxic properties reveals structural diversity and novel glycan modifications. J Biol Chem. 2018 10.1074/jbc.RA118.002971 . 29739851 PMC6028968

