# Supplementary figures and images for: Odiparcil, a potential glycosaminoglycans clearance therapy in mucopolysaccharidosis VI—Evidence from in vitro and in vivo models
Source: PLoS One. 2020 May 15;15(5):e0233032. doi: 10.1371/journal.pone.0233032 (PMC7228089; doi:10.1371/journal.pone.0233032)

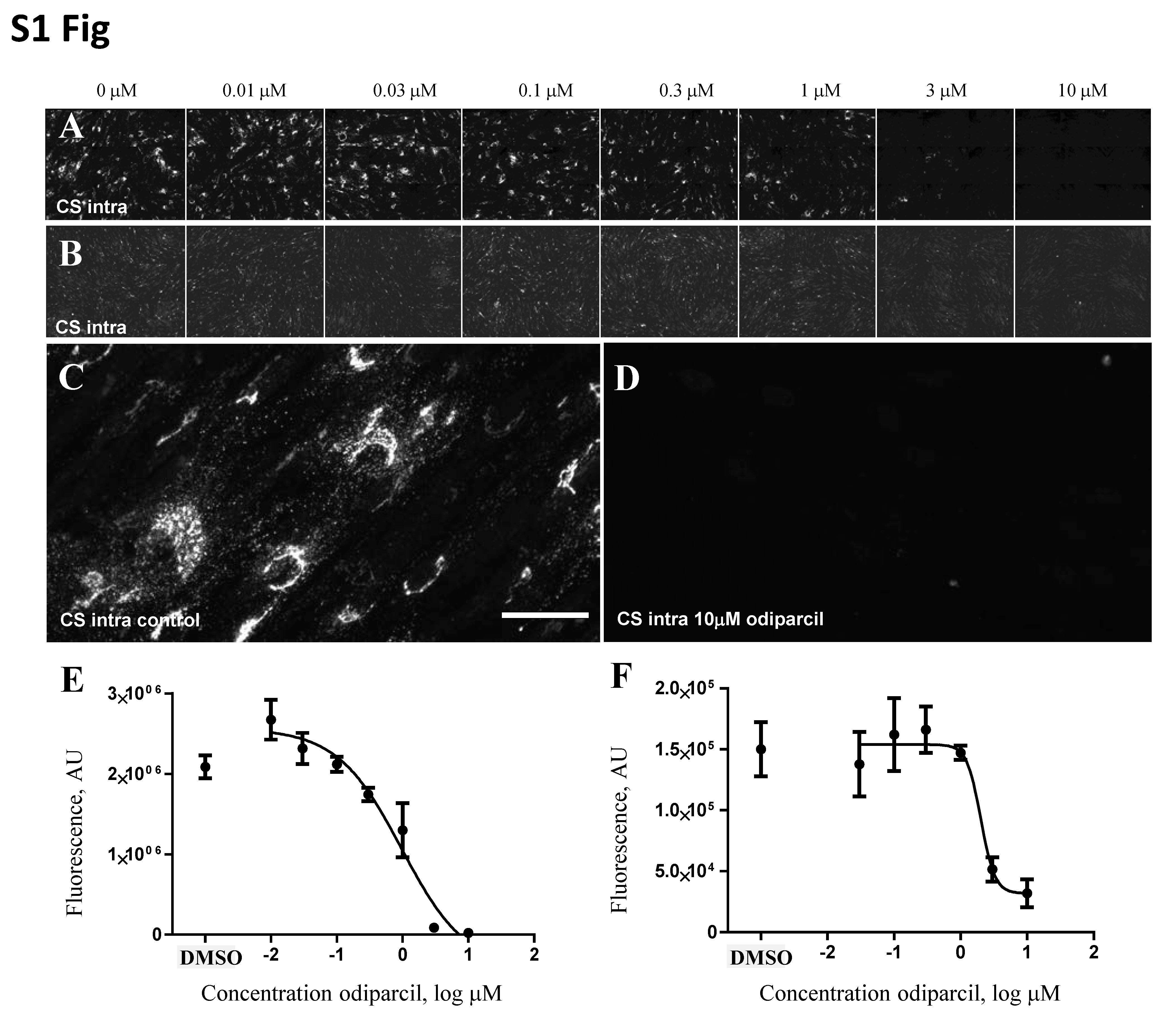

Supplement: S1 Fig — A, B. Effect of odiparcil treatment on intracellular CS in MPS VI patient fibroblasts GM02572 in growing cells culture conditions (A) and in confluent cells culture conditions (B). C, D. High magnification of intracellular CS staining in MPS VI patient fibroblasts GM02572, no odiparcil treatment (C) and 10 μM odiparcil (D). E, F. Quantification of the effect of odiparcil on the intracellular CS in MPS VI patient fibroblasts GM02572 in growing cells culture conditions (E) and in confluent cells culture conditions (F). Scale bar: C, D– 50 μm. E-G—Data represented as mean ± SEM. (TIF) [file pone.0233032.s001.tif]

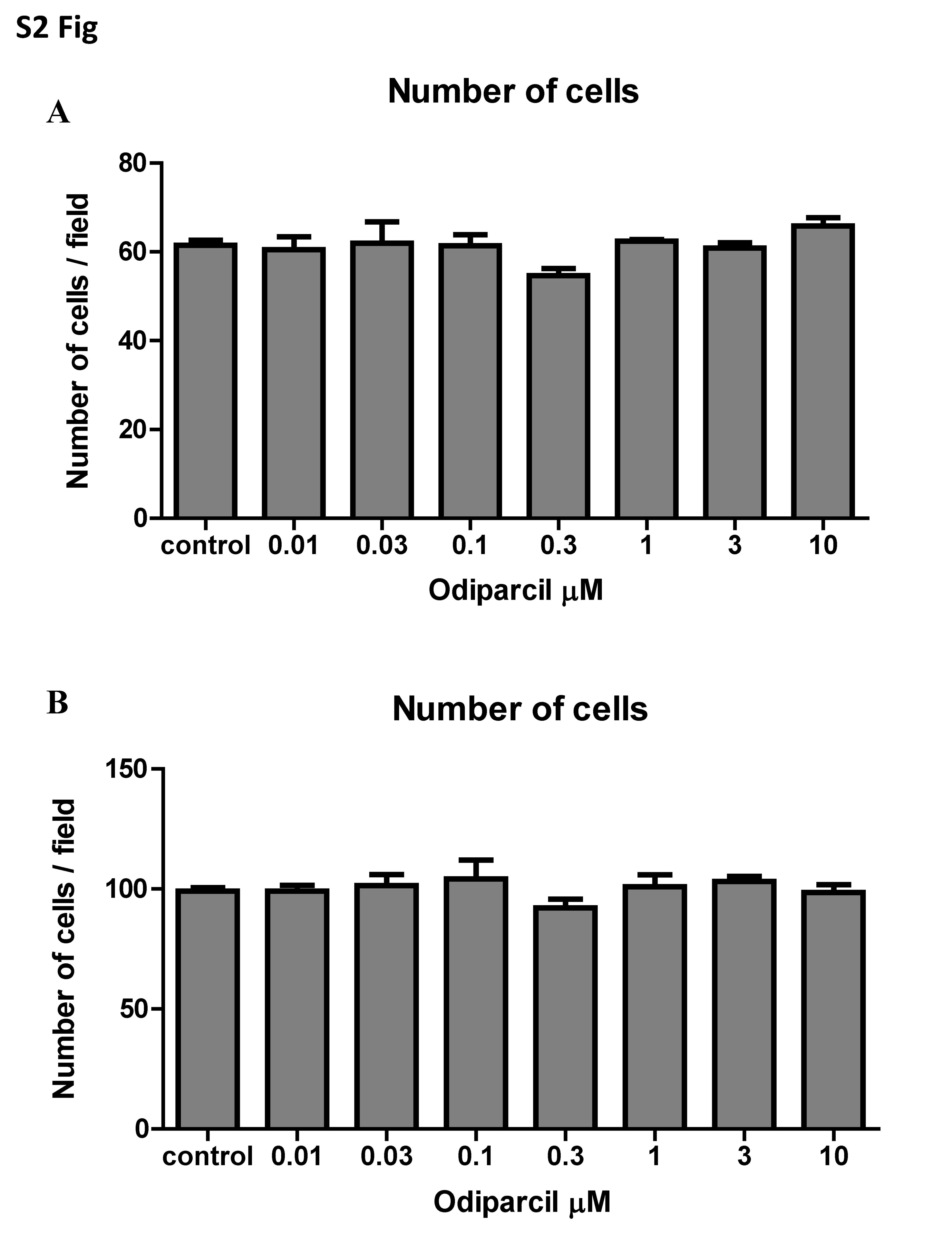

Supplement: S2 Fig — Mean number of cells from MPS VI donor GM00538 per examination field after odiparcil treatment in growing cell culture conditions (A) and in confluent cell culture conditions (B). (TIF) [file pone.0233032.s002.tif]

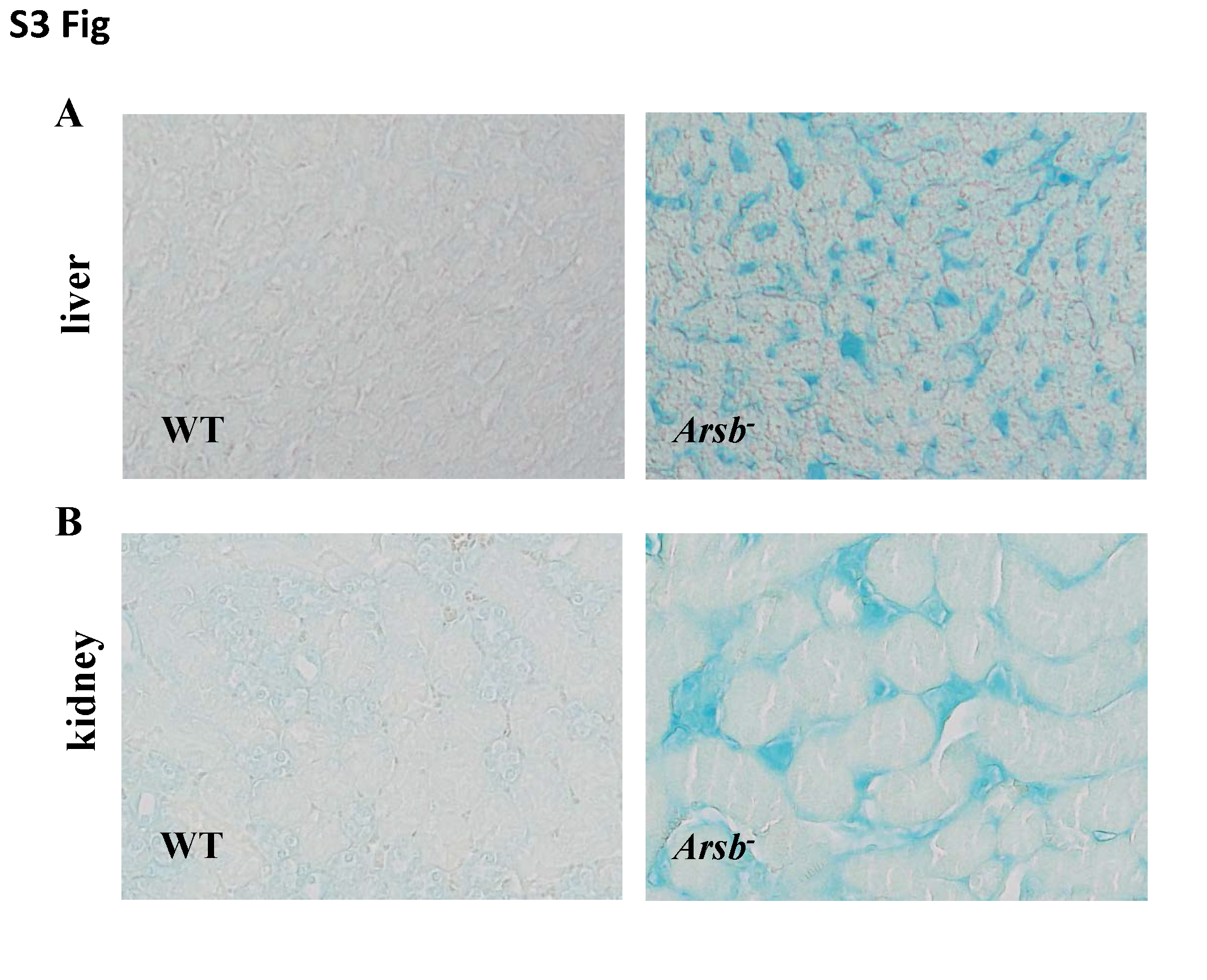

Supplement: S3 Fig — Liver (A) and kidney (B) sections stained with Alcian Blue from mice at 6 months of age, note higher levels of Alcian Blue staining in Arsb- liver and kidney. (TIF) [file pone.0233032.s003.tif]

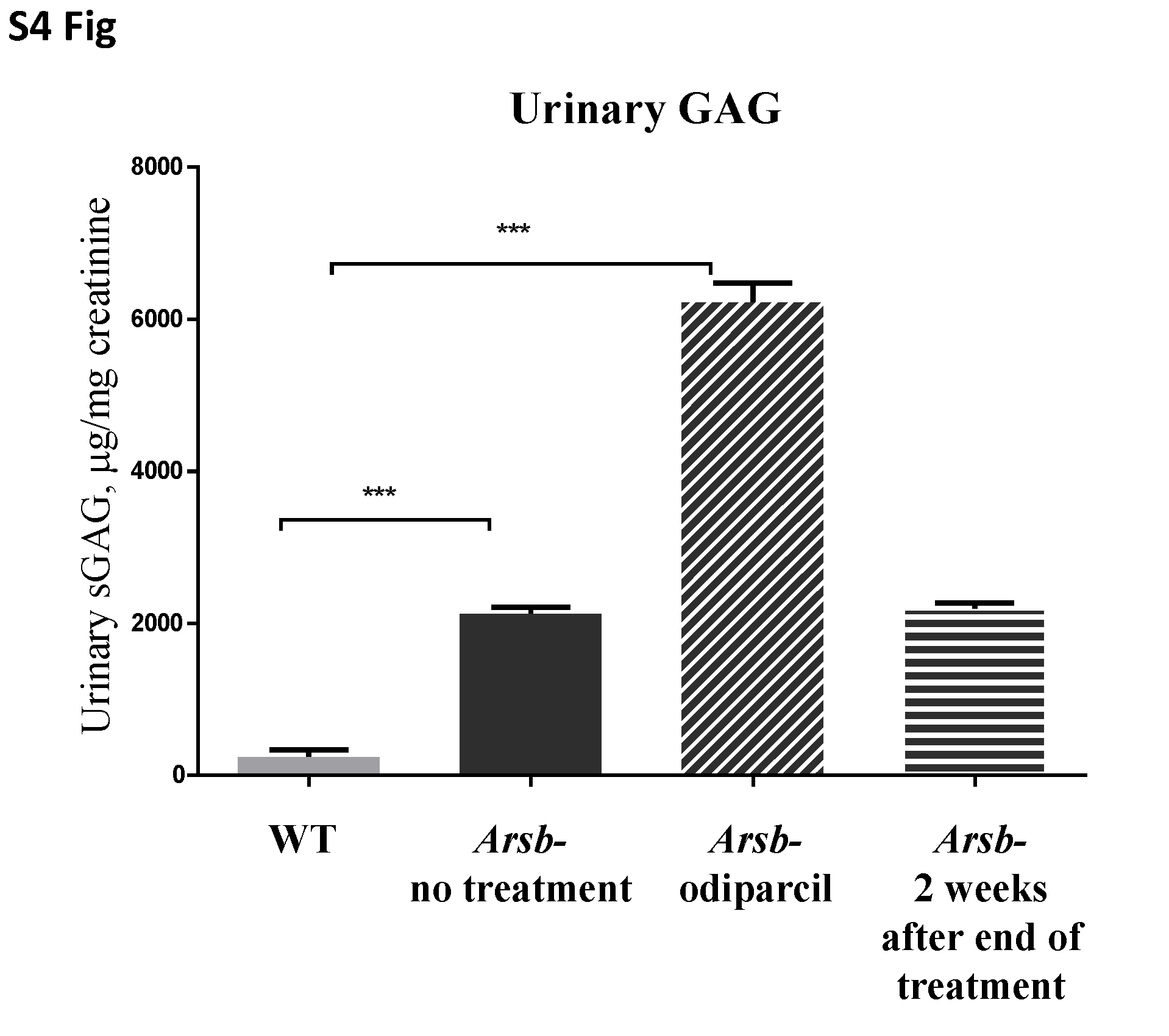

Supplement: S4 Fig — Data represented as mean ± SEM; ***: p value < 0.001. (TIF) [file pone.0233032.s004.tif]

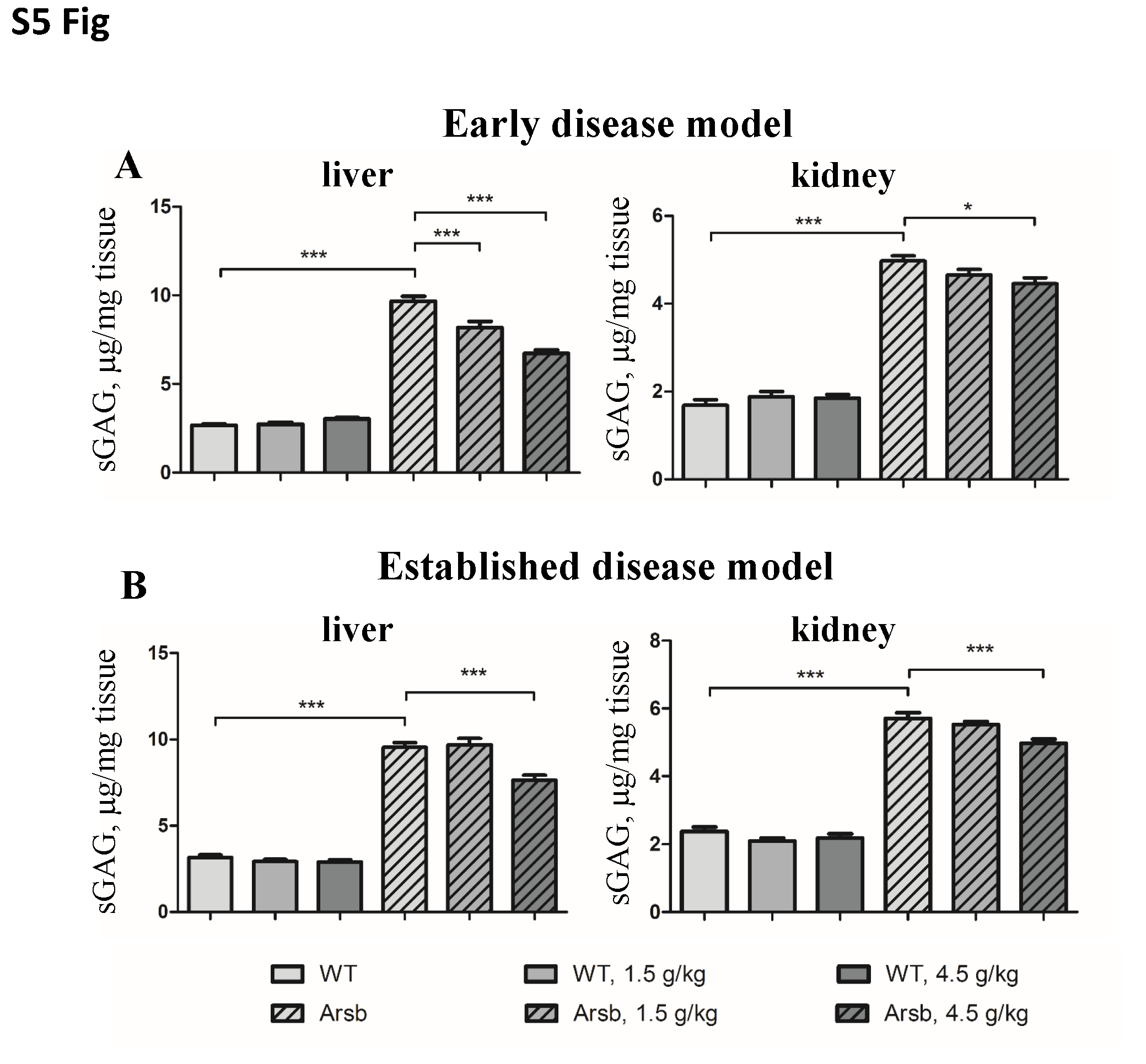

Supplement: S5 Fig — Effect of odiparcil treatment on total GAG detected by Blyscan method in liver and kidney of Arsb- mice in the early disease model (A) and in the advanced disease model (B). Data represented as mean ± SEM;*: p-value<0.05, **: p value < 0.01; ***: p value < 0.001. (TIF) [file pone.0233032.s005.tif]
