# Supplementary material for: Odiparcil, a potential glycosaminoglycans clearance therapy in mucopolysaccharidosis VI—Evidence from in vitro and in vivo models
Source: PLoS One. 2020 May 15;15(5):e0233032. doi: 10.1371/journal.pone.0233032 (PMC7228089; doi:10.1371/journal.pone.0233032)
Supplement: S1 Table — Percentage of secreted CSGAG (comprising CS and DS) and HSGAG were calculated as % from total GAG in individual separate reactions of degradation by specific enzyme (CSase ABC or Heparitinase II). That is why the sum of Mean CSGAG (%) and Mean HSGAG (%) at a given odiparcil concentration is not 100%. (DOCX) [file pone.0233032.s006.docx]

**S1 Table. Relative presence of CSGAG and HSGAG in cell culture media of BAE cells treated with odiparcil.** Percentage of secreted CSGAG (comprising CS and DS) and HSGAG were calculated as % from total GAG in individual separate reactions of degradation by specific enzyme (CSase ABC or Heparitinase II). That is why the sum of Mean CSGAG (%) and Mean HSGAG (%) at a given odiparcil concentration is not 100%.

| **Odiparcil concentration (µM)** | **Mean CSGAG (%)** | **Mean HSGAG (%)** |
| --- | --- | --- |
| 0 | 72.1 | 40.4 |
| 0.1 | 78.5 | 34.5 |
| 0.3 | 83.5 | 28.0 |
| 1 | 83.9 | 16.0 |
| 3 | 82.5 | 15.0 |
| 10 | 73.5 | 19.5 |
